# Identification and characterization of a new family of long satellite DNA, specific of true toads (Anura, Amphibia, Bufonidae)

**DOI:** 10.1038/s41598-022-18051-9

**Published:** 2022-08-17

**Authors:** Katerina Guzmán, Álvaro S. Roco, Matthias Stöck, Adrián Ruiz-García, Enrique García-Muñoz, Mónica Bullejos

**Affiliations:** 1 Departamento de Biología Experimental, Facultad de Ciencias Experimentales, Universidad de Jaén, Campus Las Lagunillas S/N, 23071 Jaén, Spain; 2 Leibniz-Institute of Freshwater Ecology and Inland Fisheries (IGB), Müggelseedamm 301, 12587 Berlin, Germany; 3 Grupo ECOBISA (RNM-300), Departamento de Biología Animal, Biología Vegetal y Ecología, Facultad de Ciencias Experimentales, Universidad de Jaén, Campus Las Lagunillas S/N, 23071 Jaén, Spain

**Keywords:** Genetics, Cytogenetics, Evolutionary biology, Evolution, Phylogenetics

## Abstract

Amphibians have some of the most variable genome sizes among vertebrates. Genome size variation has been attributed to repetitive and noncoding DNA, including satellite repeats, transposable elements, introns, and nuclear insertions of viral and organelle DNA. In vertebrates, satellite DNAs have been widely described in mammals, but few molecular studies have been carried out in amphibians. Here, we provide a detailed characterization of a new family of satellite DNA, present in all 15 examined species of the family Bufonidae. Southern-blot analysis and PCR reveal that this satellite is formed by monomers of 807 bp, is organized in tandem arrays, and has an AT-content of 57.4%. Phylogenetic analyses show that most clades exhibit species-specific variances, indicating that this satellite DNA has evolved by concerted evolution. The homogenization/fixation process is heterogeneous in Bufonidae, where the genera *Bufo* and *Bufotes* do not show species-specific differences, while populations from *Rhinella marina* exhibit population-specific changes. Additionally, variants of this satellite DNA have been identified in *Duttaphrynus melanostictus* and *R. marina*, supporting the ‘library hypothesis’ (a set, ‘library’, of satellite DNAs is shared by a species group). Physical mapping in *Bufo bufo*, *Bufo spinosus*, *Epidalea calamita* and *Bufotes viridis* provides evidence that this repetitive DNA is not dispersed in the karyotype, but accumulated in pericentromeric regions of some chromosomal pairs. This location, together with its presence in the transcriptomes of bufonids, could indicate a role in centromere function or heterochromatin formation and maintenance.

## Introduction

Satellite DNA sequences are repeated in tandem arrays, hundreds to thousands of times in the genome. They are predominantly localized in the centromeric, pericentromeric, and telomeric regions, and present the most abundant components of constitutive heterochromatin^1^. Monomers of satellite DNA accumulate mutations, but the divergence between monomers is usually low as they do not evolve independently but by concerted evolution. This involves a two-step process: (1) changes spread to other sequences (homogenization) by non-reciprocal exchanges (such as uneven crossing-over, gene conversion, rolling-circle amplification, or slippage replication), and (2) they are fixed (or eliminated) in reproductively isolated groups^2,3^. Furthermore, several sets of variants and satellite DNAs can be shared by a species group (also called a ‘library’), leading to species-specific profiles due to differential amplification and/or contractions of repetitive sequences within such a library^2,4^. Satellite DNA evolution may also be affected by other factors, such as location within heterochromatin, the reproductive biology of the species, or by its population size^2^.

Repetitive DNA research has mostly been based on analyses of randomly cloned monomers and short multimers, obtained after digestion of genomic DNA and mapping by fluorescent in situ hybridization (FISH)—techniques, which are still used today^5^. The application of whole genome sequencing to satellite DNA research has been limited to graph-based clustering analysis and manual annotation, and only recently the availability of long read technology overcomes the problems posed by the assembly of long repetitive motives.

Satellite DNA is no longer considered ‘junk’. These sequences have been implicated in genome regulation^6^, transcription^7^, centromere identity^8^, genome architecture^9,10^, and other important cell functions^11^. Applications to evolutionary studies are also interesting, as they can provide information on the divergence between species^12^ and about the existence of possible mechanisms of reproductive isolation, triggered, for example, by the diversification of centromeric repetitive DNA sequences^13^. However, even with recognizing the functional importance of satellite DNA, the structural and functional knowledge about these sequences is still limited.

Amphibians are the extant vertebrate class with the largest range of genome sizes^14^. The differences of DNA-content and chromosome size in this group were not attributed to the visible presence of constitutive heterochromatin^15^. In the past, numerous studies about the re-association kinetics of satellite DNA and chromosomal organization in amphibians concluded that repetitive sequences play an important role in chromosome size differences in anurans (Supplementary Bibliography, references 1–6). Scarce information about satellite DNA obtained by genome-wide analysis confirms the high amount of satellite DNA present in amphibian genomes (e.g. 15.7% in *Proceratophrys boiei*^16^).

Bufonidae presents the fourth largest systematic amphibian family, with 52 genera and 634 known species^17^ (638 as reported in Amphibian Species of the World^18^). This group has been subject to many phylogenetic studies, although its systematics is challenging. Along with Pipidae and Ranidae, Bufonidae are among the best-studied amphibian families. However, molecular and genetic data about genome organization in this group remain insufficient. The only information available about repetitive DNA sequences is restricted to a single 420 bp sequence (AY585341) from a satellite DNA (pBv) isolated from a single green toad (*Bufotes viridis* species group), probably present in several Bufonidae genera (*Bufo*, *Bufotes*, and probably *Werneria* and *Wolterstoffina*)^19^.

Repetitive sequences in amphibians are related to/derived from transposons, like the highly repetitive DNA R.e/Tc1 in Ranidae (*Pelophylax esculentus*)^20^, from 5S rDNA, like the PcP190 satellite of Hyloidea^21^, or telomeric sequences, like the ITRs in Pipidae (genus *Xenopus*)^22^. Initially, most amphibian satellite DNAs were described in Caudata, primarily in *Triturus*, and in other genera or families (Supplementary Bibliography, references 7–12). In Anura, satellite DNA has been widely studied in *Xenopus*, *Rana*, *Pelophylax*, and Discoglossidae (Supplementary Bibliography, references 13–28).

Here, we identify a new satellite DNA-family, present in several species of Bufonidae. This satellite DNA has a long monomer size of 807 bp, organized in tandem arrays. Phylogenetic analysis suggests that it has evolved according to the concerted evolution model. Different homogenization/fixation rates are observed in different species. Accordingly, the homogenization of sequences appears unfinished in some species, while we observed differences between populations in other taxa. Furthermore, variants supporting the ‘library hypothesis’ are also present in some species. The accumulation of these repetitive sequences in pericentromeric regions of some chromosomal pairs in *Bufo bufo*, *Bufo spinosus*, *E. calamita*, and *Bufotes viridis*, together with the evidence of their transcription after the analysis of bufonid transcriptomes, may implicate them in centromere function or heterochromatin formation and maintenance.

## Results

### Identification, cloning and characterization of a repetitive DNA family in species of the *Bufo bufo* group

**Figure 1:**
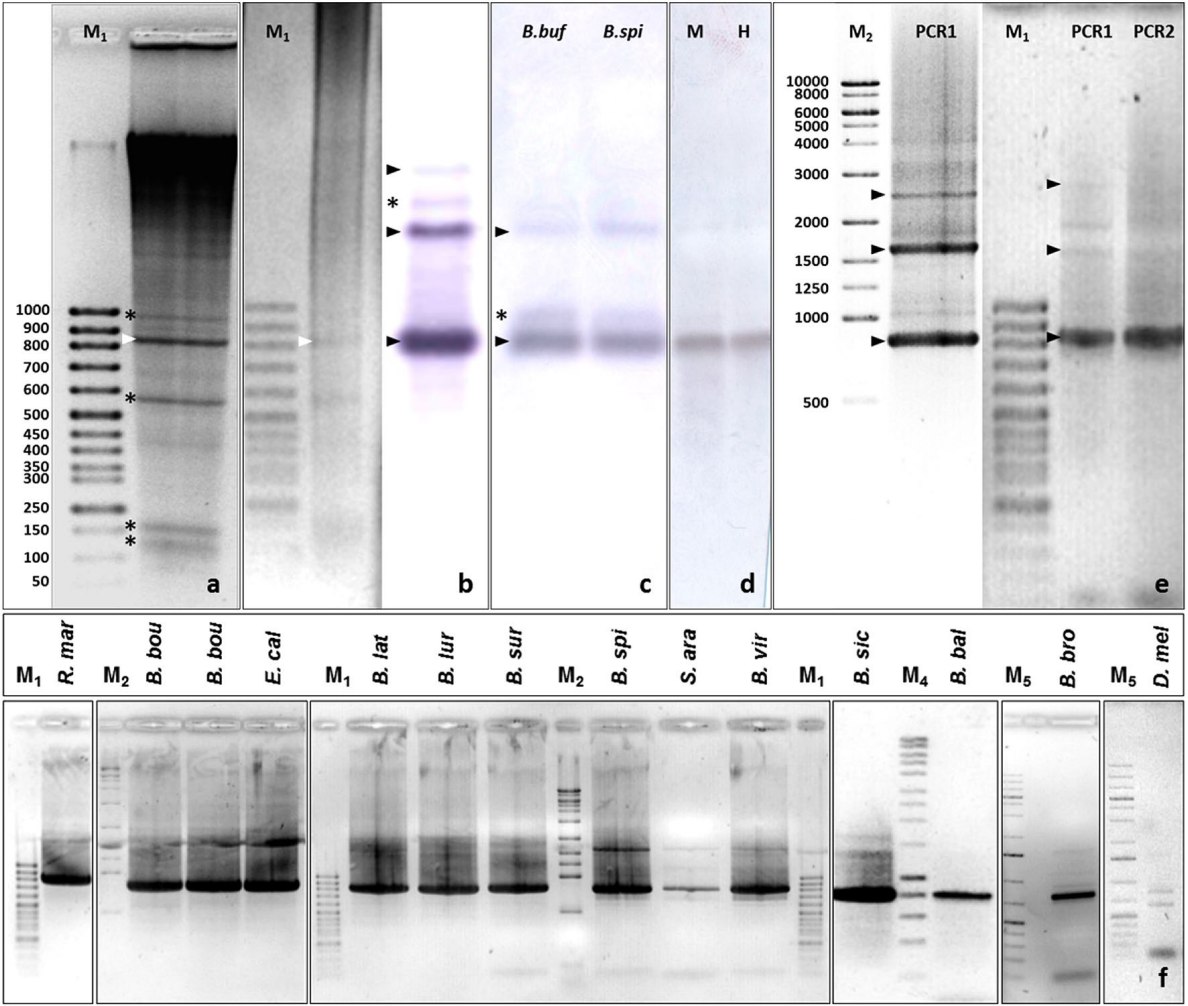
(**a**) Reversed image of the agarose gel electrophoresis of a genomic DNA sample from *Bufo bufo* (Bologna, Italy) digested with BamHI. Intense bands indicate different repetitive DNA families (*). The white arrowheads point to the position of the analysed band, that was eluted from the gel and cloned into BamHI-digested pCU19 vector. (**b**) Inverted image of the agarose gel electrophoresis of a DNA sample from *Bufo bufo* H2 (Jaén, Spain) digested with BamHI and blotted onto a nylon membrane, hybridized with the dig-labelled BamHI-800 bp band. A ladder-like pattern is observed (800, 1600, 2000 and 2400 bp). (**c**) Southern-Blot of BamHI-digested genomic DNA from *Bufo bufo* (*B. buf*: sample from Bologna, Italy) and *Bufo spinosus* (*B. spi*: sample from Jaén, Spain). (**d**) Southern-Blot of BamHI-digested genomic DNA from male (M) and female (F) *Bufo spinosus* (both from Jaén). (**e**) PCR using primers Bbu-BamHI-800-F1/R1 (PCR1) or Bbu-BamHI-800-F2/R2 (PCR2) (reversed image) with genomic DNA from *Bufo spinosus*. Black arrowheads point to 800, 1600, and 2400 bp bands; the bands labelled with (*) in B and C are explained by the star activity of BamHI restriction enzyme, as this band is not always observed. (**f**) PCR amplification of BamHI-800 sequences in other species of the Bufonidae family. Code used for the name of the species: *Bufotes boulengeri* (*B. bou*), *E. calamita* (*E. cal*), *Bufotes luristanicus* (*B. lur*), *Bufotes surdus* (*B. sur*), *Bufotes viridis* (*B. vir*), *B. bufo* (*B. buf*), *R. marina* (*R. mar*), *Bufotes latastii* (*B. lat*), *S. arabica* (*S. ara*), *Bufotes siculus* (*B. sic*), and *Bufotes balearicus* (*B. bal*), *Ba. brongersmai* (*B. bro*), and *D. melanostictus* (*D. mel*). Original gels/blots are presented in Supplementary Fig. S7. For a detailed description of molecular weight markers see Supplementary Table S9.

Genomic DNA from one adult *Bufo bufo* female from Bologna (Italy) was digested with the restriction endonuclease BamHI. Electrophoresis of digested DNA showed several intense bands, indicating the presence of several repetitive DNAs (Fig. 1a). The band of ca. 800 bp was excised, purified, labelled and used as a probe in a Southern blot analysis. Positive bands of about 800 bp, 1600 bp, and 2400 bp revealed a “ladder”-like pattern of bands typical of repetitive DNA, organized in tandem arrays (Fig. 1b). The same band pattern was observed when genomic DNA from *Bufo spinosus* (Jaén, Spain) was analysed (Fig. 1c). Except for the bands due to star activity of BamHI, no variation in the banding pattern of Southern-blot experiments was found when DNAs from both species or both sexes were analysed (Fig. 1c,d).

The band from *Bufo bufo* of about 800 bp was cloned in a dephosphorylated BamHI-digested pUC19 vector (Fermentas, Vilnius, Lithuania). Recombinant plasmids were purified and sequenced. The sequences from six clones were aligned and compared with each other. Their size ranged between 810 and 811 bp, except for one clone with a small deletion of 22 bp (789 bp) and an incomplete clone of 492 bp (312 bp missing from the 5′-end) that was not used in the analysis. The average A + T content was 57.5%, and no direct or inverted internal repeats were observed, except for a small fragment of 20 bp, repeated 1.9 times (positions 13–50).

Based on these sequences, two primer pairs with opposite orientations were designed (Supplementary Fig. S1). PCRs with both primer pairs gave the same banding pattern: 800 bp, 1600 bp, and 2400 bp (Fig. 1e), indicating that BamHI-800 sequences are monomeric units of a repetitive DNA family, organized in tandem arrays. This was further confirmed as positive clones obtained from the 1600 bp PCR band are dimers of 800 bp monomer units, separated by target sites for BamHI.

Using BamHI-800-specific primers (Supplementary Fig. S1), BamHI-800 sequences were amplified from different DNAs from *Bufo spinosus* specimens. Cloning and sequencing these amplicons gave five sequences from a *Bufo spinosus* sample from Morocco and 38 sequences from several *Bufo spinosus* from Spain (Jaén) (Table 1 and Supplementary Table S1). Incomplete clones (182 and 655 bp) were not analysed (details: Supplementary Table S1).

**Table 1:** Samples analysed in this study.

Family	Species	2n	Location	SEX	Band	PCR	N
Bufonidae	*Bufo bufo*^1^	22	Italy (Bologna)	F	6 (1*)		6
Bufonidae	*Bufo spinosus*^1^	22	Morocco			5	5
Bufonidae	*Bufo spinosus*	22	Spain (Jaén)			5	5
Bufonidae	*Bufo spinosus*	22	Spain (Jaén)			5	5
Bufonidae	*Bufo spinosus*	22	Spain (Jaén)	M		7 (D)	7
Bufonidae	*Bufo spinosus*	22	Spain (Jaén)	M&F		26 (2*)	1
Bufonidae	*Bufotes viridis*	22	Greece (Crete)	M		10	10
Bufonidae	*Bufotes boulengeri*^1^	22	Morocco			13	13
Bufonidae	*Bufotes boulengeri*	22	Morocco			14	14
Bufonidae	*Bufotes siculus*	22	Italy (Sicily)	M/F		4	4
Bufonidae	*Bufotes balearicus*	22	Italy (Sicily)	M/F		4	4
Bufonidae	*Bufotes latastii*	22	Pakistan			8 (1*)	8
Bufonidae	*Bufotes luristanicus*	22	Iran			14 (1*)	14
Bufonidae	*Bufotes surdus*	22	Iran			13	13
Bufonidae	*Epidalea calamita*	22	Spain (Jaén)	M	11 (2*)	6	17
Bufonidae	*Epidalea calamita*	22	Spain (Jaén)		2		2
Bufonidae	*Epidalea calamita*	22	Spain (Jaén)			20 (D)	20
Bufonidae	*Rhinella marina*^1^	22	Australia (Queensland)			7 (2*)	7
Bufonidae	*Sclerophrys arabica*	20	Yemen			19	19
Bufonidae	*Duttaphrynus melanostictus*	22	Nepal			14	14
Bufonidae	*Barbarophryne brongersmai*	22	Morocco			12 (1*)	12
Alytidae	*Discoglossus galganoi*	28	Spain (Andalucía)			–	–
Ranidae	*Pelophylax perezi*	26	Spain (Jaén)			–	–
Pipidae	*Xenopus tropicalis*	20	Lab strain (Nigeria)			–	–
TOTAL					225 (10*)		200

For each sample, the following information is provided: anuran family, species name, chromosome number, geographical origin of the sample, sex (if known), number of sequences obtained from digested DNA (band), number of sequences obtained from specific PCR (PCR), and number of sequences used in the phylogenetic analysis (N). (D): monomer sequences from dimers of ca. 1600 bp; (*): incomplete fragments. ^1^Samples from tissue donations.

The alignment of all *Bufo bufo* group sequences (Supplementary Fig. S2) resulted in a consensus sequence of 819 bp (deletions from 1 to 28 bp were observed in some sequences). This repetitive motif has an average A + T composition of 57.4%, while the average R (transition/transversion ratio) was 0.85. When *Bufo bufo*-group BamHI-800 sequences are grouped according to species (*Bufo bufo* and *Bufo spinosus*), population (Bologna, Spain and Morocco), sex (male and female), or experimental procedure ((i) bands: obtained from BamHI digested genomic DNA fragments, (ii) PCR products with F1/R1 primers, (iii) PCR products with F2/R2 primers), the differences in the average genetic distances within and between groups were not significant (Supplementary Table S2, Supplementary Fig. S3).

### BamHI-800 sequences in other amphibian species

To evaluate the presence of this satellite DNA in other amphibian species, we carried out Southern-Blot analysis on BamHI-digested DNAs from several anuran families using BamHI-800 sequences from *Bufo bufo* as a probe. This analysis comprised six species of Bufonidae (*Bufotes viridis*, *Bufotes boulengeri*, *Bufotes luristanicus*, *Bufotes surdus*, *Epidalea calamita*, and *Rhinella marina*) and three species from other anuran families *Discoglossus galganoi* (Alytidae), *Xenopus tropicalis* (Pipidae), and *Pelophylax perezi* (Ranidae) (Table 1). Interestingly, positive hybridization signals corresponding to BamHI-800 sequences were only observed in Bufonidae (Supplementary Fig. S4a), with all species showing the same ladder-like pattern: ca. 800 bp, 1600 bp, and 2400 bp, corresponding to monomers, dimers, and trimers.

The presence of BamHI-800 repetitive DNA sequences was also examined by PCR in 12 bufonid toad species and in three species of other amphibian families (Table 1 for sample details). Amplification with both primer pairs (Bbu-BamHI-800-F1/R1 and Bbu-BamHI-800-F2/R2) was positive only in Bufonidae. Again, identical patterns corresponding to monomer and sometimes dimer and trimer repeats of 800 bp units were observed in most species (Fig. 1f). Small differences to this common pattern were observed in some cases: (1) less intense bands in *Sclerophrys arabica* and *Duttaphrynus melanosticus*, and (2) some extra bands in *S. arabica*, *D. melanosticus* and *Barbarophryne brongersmai* (Fig. 1f and Supplementary Fig. S4b). The monomer band was cloned and sequenced in each bufonid species in Table 1 (Supplementary Table S1 for details about clones/sequences). Of note, some clones from *D. melanosticus* (5 out of 14) had inserts longer than 800 bp (up to 1100 bp) that were difficult to sequence. The alignment of the sequences obtained (three partial and two complete sequences) revealed the presence of an insertion at the same position in all the clones (Supplementary Fig. S5a). In addition, the inserted sequences had long palindromes (Supplementary Fig. S5a,b) that form long hairpin structures when the secondary structure is predicted (Supplementary Fig. S5c). The existence of this palindromic region produces artefacts in PCRs from these clones when universal primers (M13 (− 20) and M13 Reverse) are used (Supplementary Fig. S5d). These extra bands of smaller size could explain the difficulty experienced to sequence these clones. The inserted region of clone Dmel PCR2-1 23 INS (303 bp) was used in BLASTN searches on DNA databases (NCBI, nr/nt). Hits in several *Bufo bufo* and *Bufo gargarizans* genes/sequences were identified, although none was related to transposable elements or repetitive sequences. For further analysis of these sequences, the inserted sequence (red box in Supplementary Fig. S5a) was removed.

### BamHI-800 sequences from genetic databases

A BLASTN search on DNA databases (NCBI, nr/nt) produced only three hits: a predicted ncRNA from *Bufo bufo* (XR_005776185.1, 69% identity) and two predicted transcript variants from *Bufo gargarizans* (XR_006388564.1, XR_006388563.1, 66% identity). BLASTN searches were also performed against all amphibian genome assemblies available on NCBI (Supplementary Table S3). Exclusively bufonid genome assemblies displayed significant hits: *Bufo bufo* (GCF_905171765.1), *Bufo gargarizans* (GCA_014858855.1), and *R. marina* (GCA_900303285.1). Genomes from two Dendrobatidae and one Leptodactylidae species, the closer outgroups available, did not show BLASTN hits.

BLASTN searches against the *Bufo bufo* genome produced 5768 hits. Sequence numbers according to their chromosome/unknown localization are shown in Supplementary Table S4. A selection of 1376 sequences without deletions at the 5’- or 3’-ends was further analysed. They have an average monomer size of 809 bp, an A + T content of 57.1%, and identity ranges between 100 and 78% (Table 2).

**Table 2:** Subfamilies of repetitive BamHI-800 and number of sequences found for each one.

Species	Variant	N	Size	T. N	NC	NV-I	NV-SNP	%AT	R	%Identity	Distance
*Bufo bufo*		6	807	818	712	34	65	57.5	0.92	98.2–90.5	0.0176–0.0704
*Bufo spinosus*		23	809	813	586	73	153	57.4	0.83	97.9–90.0	0.0189–0.0869
*Bufotes viridis*		10	810	812	678	38	95	56.9	0.68	98.8–91.8	0.0112–0.0824
*Bufotes boulengeri*		27	811	815	534	96	182	56.8	0.63	98.5–90.3	0.0150–0.1056
*Bufotes siculus*		4	811	812	783	10	18	57.1	0.46	99.6–97.4	0.0025–0.0265
*Bufotes balearicus*		4	811	813	741	9	61	57.3	0.86	96.6–95.0	0.0317–0.0518
*Bufotes latastii*		8	812	827	643	69	100	56.7	0.88	96.3–88.1	0.0330–0.1122
*Bufotes luristanicus*		14	812	825	640	71	101	56.4	0.83	100.0–89.6	0.0000–0.0998
*Bufotes surdus*		13	814	831	638	66	121	56.6	0.95	99.8–90.0	0.0012–0.1134
*E. calamita*		39	812	821	596	72	147	58.0	0.86	100.0–94.4	0.0000–0.0530
*R. marina*		7	810	812	713	28	70	57.7	1.04	100.0–93.7	0.0000–0.0658
*S. arabica*	V1 + V2	19	789	832	524	176	125	57.2	1.08	100–75.3	0.0000–0.2200
	V1	14	807	832	573	139	108	57.6	1.22	100.0–78.6	0.0000–0.2143
	V2	5	739	832	657	1	69	56.0	1.12	99.8–82.4	0.0014–0.1020
*D. melanosticus*	V1 + V2	14	806	824	542	213	66	59.2	1.03	100.0–70.2	0.0000–0.3699
	V1	5	798	824	795	3	1	56.3	1.50	100.0–99.2	0.0000–0.0050
	V2	9	811	824	682	29	100	60.8	1.34	99.3–89.9	0.0075–0.1108
*Ba. bronguersmai*		13	772	790	592	66	128	56.6	0.94	99.2–83.0	0.0064–0.1580
Bufonidae (all)	V1 + V2	200	806	897	108	648	111	57.3	0.92	100.0–58.4	0.0000–0.4787
	V1	186	808	881	120	604	130	57.2	0.93	100.0–65.5	0.0000–0.3939
*Bufo bufo* (*)		1370	809	822	11	761	40	57.1	1.03	100.0–78.2	0.0000–0.2031
*Bufo gargarizans* (*)		953	809	814	21	735	56	57.3	0.75	100.0–71.7	0.0000–0.3400
*R. marina* (*)	V1 + V2	15	797	811	344	225	212	57.2	0.86	100.0–69.0	0.0000–0.2902
	V1	8	800	811	554	90	167	56.9	1.10	93.3–78.4	0.0512–0.1895
	V2	7	795	811	492	117	200	57.6	0.84	100.0–72.2	0.0000–0.2790
Bufonidae (all + 30*)		230	807	893	86	684	97	57.4	0.92	100.0–58.2	0.0000–0.4787

For each group of sequences with N: number of sequences; Size: average size of the sequences; T.N: total number of sites; NC: number of conserved positions; NV-I: number of variable informative sites; NV-SNP: number of single nucleotide polymorphisms; R: transitional pairs/transversional pairs. (*) Sequences from genome assemblies.

BLASTN searches against the *Bufo gargarizans* genome produced 2031 hits. The number of sequences according to their chromosome/unknown localization is shown in Supplementary Table S4. A selection of 953 sequences without deletions at their 5’- or 3’-ends was further analysed. They show an average monomer size of 809 bp, an A + T content of 57.3%, and identity ranges between 100 and 71.7% (Table 2).

Finally, BLASTN searches against the *R. marina* genome produced 226 hits. No chromosome level assembly is available for this species. A selection of 15 sequences without deletions at their 5’- or 3’-ends was further analysed. Their average monomer size is 797 bp, an A + T content of 57.2%, and identity ranges between 100 and 69% (Table 2).

### Analysis of BamHI-800 sequences

In total, 230 BamHI-800 sequences from 15 bufonid species were analysed (200 cloned sequences and 30 sequences retrieved after BLASTN on genome assemblies of *Bufo bufo*, *Bufo gargarizans*, and *R. marina*; first ten hits per species).

Percentages of identity (Supplementary Table S5A–C), pairwise distances between sequences (Supplementary Table S6A,B) and visual observation of the alignment (Supplementary Table S7) revealed two types of monomers (variants) in *D. melanosticus*, *R. marina*, and *S. arabica*. According to this, in these species, we considered as variant 1 (V1) those sequences with higher sequence identity with *Bufo bufo* group sequences, and as variant 2 (V2) those with lower identity values. V1 and V2 sequences were analysed together and independently.

The comparative analysis of all bufonid BamHI-800 sequences, including the results of the analysis of sequences grouped by species and variants, is shown in Table 2. The average monomer size is 807 bp (814 to 790 bp), and the average A + T% content is 57.4% (56.0% to 60.8%, both in V2 variants). Within species, this satellite DNA shows lower genetic distances when V1 or V2 sequences are analysed independently (between 0.0000 and 0.2143, Supplementary Table S6A and diagonal in Supplementary Table S6B). However, in pairwise comparisons between all the sequences, genetic distances increase to 0.4787 when V2 sequences from *D. melanostictus* are compared to *S. arabica* V1 (Supplementary Table S6A,B). Higher genetic distances were observed in pairwise comparisons that include sequences from *Ba. brongersmai*, *D. melanosticus*, *R. marina*, and *S. arabica*. However, *S. arabica* V2 sequences are closer to *S. arabica* V1 sequences than to V2 sequences from *R. marina* and *D. melanosticus*. A close look to the alignment of these sequences (Supplementary Table S7) reveal that the increased distances between V1 and V2 variants from *S. arabica* is mainly due to the presence of long deletions in V2 sequences, and not to a high number of nucleotide differences between them.

Overall genetic distance for bufonid BamHI-800 sequences is 0.2059 ± 0.0081. Island endemic *Bufotes siculus* from Sicily shows the lowest intra-species distance: 0.0206 ± 0.0038, while the highest distances are observed in species with sequence variants: *D. melanosticus* (0.1913 ± 0.0139), *R. marina* (0.1691 ± 0.0091), and *S. arabica* (0.1313 ± 0.0085) (Supplementary Table S6B). When sequences variants are considered, the lowest intra-species distance is in variant 1 (V1) from *D. melanosticus* (0.0028 ± 0.0014), while the highest is in variant 2 (V2) from *S. arabica* (0.1101 ± 0.0076).

The highest inter-specific distance is observed between *S. arabica*–*D. melanosticus* (0.3765 ± 0.0238) and *S. arabica* and *Ba. brongersmai* (0.3485 ± 0.0221), while the lowest distances are between *Bufotes siculus*–*Bufotes boulengeri* (0.0421 ± 0.0037) and *Bufotes siculus*–*Bufotes balearicus* (0.0423 ± 0.0046) (Supplementary Table S6C). When net distances were considered, the highest distances are between *S. arabica*—*Ba. brongersmai* (0.2462 ± 0.0209) and *S. arabica*–*Bufotes siculus* (0.2340 ± 0.0203), while the lowest distances were between *Bufotes latastii*–*Bufotes luristanicus* (0.0015 ± 0.0006) and *Bufotes latastii*–*Bufotes surdus* (0.0022 ± 0.0007) (Supplementary Table S6D).

**Figure 2:**
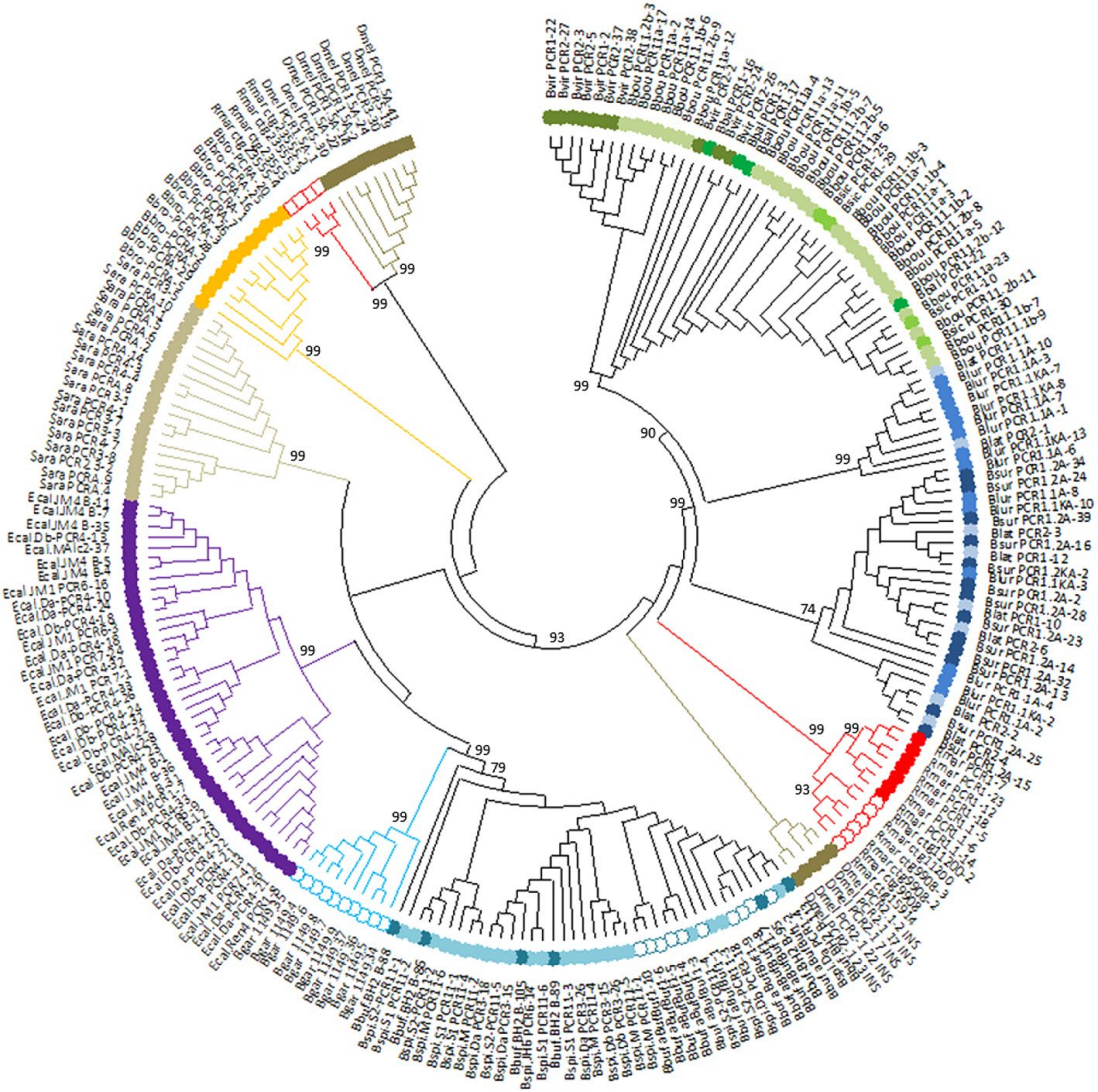
Circular tree for all BamHI-800 sequences using Maximum Likelihood and the Tamura 3-model of sequence evolution^73^. A discrete Gamma distribution was used to model evolutionary rate differences among sites (5 categories (+ G, parameter = 2.7852)). The tree with the highest log likelihood (-22,314.97) is shown. The tree is drawn to scale, with branch lengths measured in the number of substitutions per site. The percentage of trees in which the associated taxa clustered together is shown next to the main branches (values lower than 75% have been omitted). This analysis involved 230 nucleotide sequences and 746 positions in the final dataset (all positions with less than 95% site coverage were eliminated, and ambiguous bases were allowed at any position (partial deletion option)). Species names abbreviations and colour codes at branch leafs as in Fig. 1 and Supplementary Table S1.

**Figure 3:**
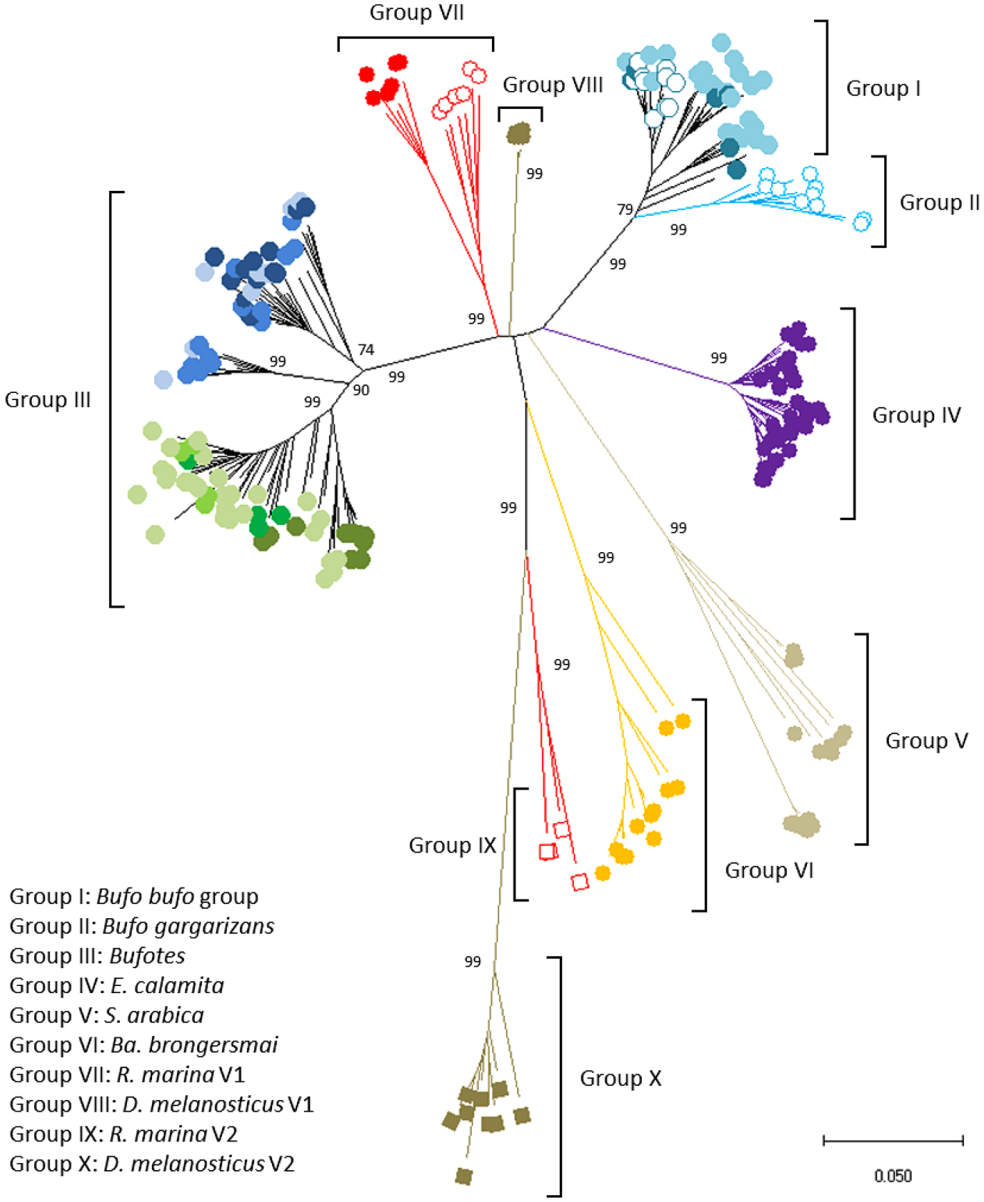
Radiation tree for BamHI-800 sequences (this work and NCBI) showing species- and genus-specific groups. The phylogenetic history was inferred as in Fig. 1. Colour codes at branch leafs as in Supplementary Table S1.

BamHI-800 sequences from 15 bufonid species were used to generate a maximum-likelihoood tree (Fig. 2). The tree contains ten major groups with bootstrap support values of 99% (Fig. 3, detailed subtrees: Supplementary Fig. S6). In each major group, sequences are clustered by species or genera (Table 3).

**Table 3:** Subfamilies/groups of repetitive DNA BamHI-800.

Genus	Species	I	II	IIIa	IIIb	IIIc	IV	V	VI	VII	VIII	IX	X
*Bufo*	*bufo*	6 (10)											
	*spinosus*	23											
	*gargarizans*		(10)										
*Bufotes*	*viridis*			10									
	*boulengeri*			27									
	*siculus*			4									
	*balearicus*			4									
	*latastii*				2	6							
	*luristanicus*				7	7							
	*surdus*					13							
*Epidalea*	*calamita*						39						
*Sclerophrys*	*arabica*							19					
*Barbarophyne*	*brongersmai*								13				
*Rhinella*	*marina*									7 (6)		(4)	
*Duttaphrynus*	*melanostictus*										5		9

Number of sequences found in each group and examined species of Bufonidae. In parenthesis are indicated the number of sequences from assembled genomes included in the analysis.

Sequences from the genus *Bufo* form two groups: group I includes sequences from *Bufo bufo* and *Bufo spinosus*, while group II consists exclusively of *Bufo gargarizans*. Within *Bufotes*, the sequences form three distinct groups: subgroup IIIa, consisting of *Bufotes viridis*, *Bufotes boulengeri*, *Bufotes siculus*, and *Bufotes balearicus*; subgroup III.b, formed by *Bufotes latastii* and *Bufotes luristanicus*; and subgroup III.c, formed by *Bufotes latastii*, *Bufotes luristanicus* and *Bufotes surdus*. Finally, sequences from *E. calamita*, *S. arabica*, and *Ba. brongersmai* are included in different groups (IV, V and VI respectively), while sequences from *R. marina* and *D. melanosticus* are split in two groups in each species: groups VII and VIII include V1 sequences, and groups IX and X are for V2 sequences.

According to Fig. 3, BamHI-800 in Bufonidae is organized into eight species-specific subfamilies (*Bufo gargarizans*, *E. calamita*, *S. arabica*, *Ba. brongersmai*, *R. marina* V1, *D. melanosticus* V1, *R. marina* V2, and *D. melanosticus* V2 from groups II, IV, V, VI, VII, VIII, IX, and X respectively); and two genera-specific subfamilies (*Bufo bufo* group and *Bufotes* from groups I and III). The sequences included in the subfamilies IX and X are distant variants (V2) of the V1 sequences in the corresponding species (*R. marina* and *D. melanosticus* respectively). In the tree, the same group (V) contains V1 and V2 sequences from *S. arabica*.

No other BamHI-800 variant has been identified besides V2 sequences from *R. marina*, *D. melanosticus*, and *S. arabica* (BLASTN searches with V2 sequences in genome assemblies from bufonids do not produce hits different from V1 sequences).

Some other interesting features in BamHI-800 tree are: (1) sequences from *Bufo gargarizans* form a group and cluster with those of the *Bufo bufo* group; (2) all *Bufo bufo* species group sequences (from Italy (*Bufo bufo*), Spain (*Bufo spinosus*), Morocco (*Bufo spinosus*), and UK (*Bufo bufo* sequences retrieved from NCBI)) appear intermixed in their group, without significant differences between them (Supplementary Fig. S3); (3) sequences in group IV (*R. marina* V1) split into two separate groups (V1 sequences obtained in this work and V1 from NCBI) (Figs. 2, 3; Supplementary Fig. S6).

### FISH analysis

**Figure 4:**
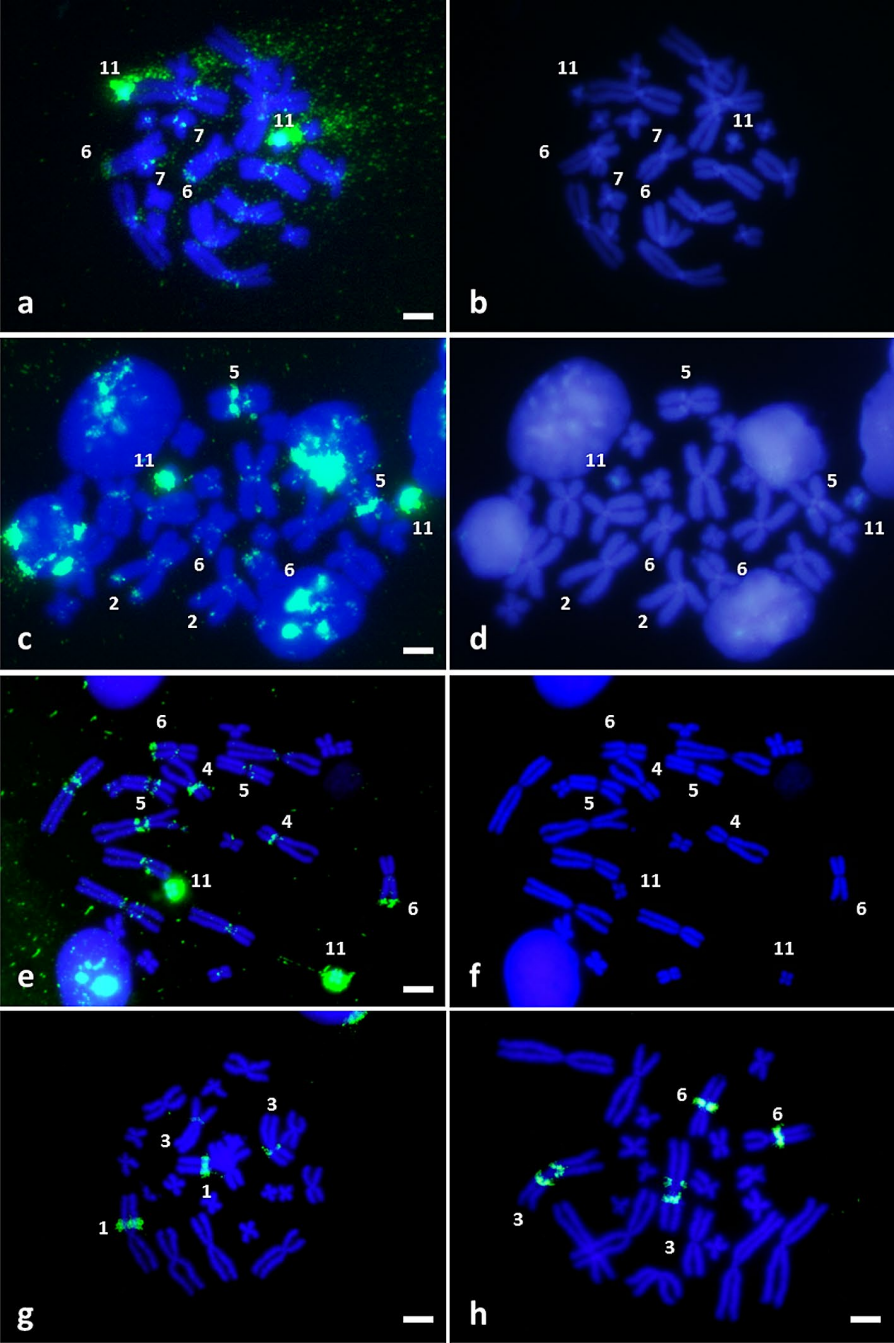
FISH on metaphase chromosomes from *Bufo bufo* (**a**,**b**); *Bufo spinosus* (**c**–**f**); *Epidalea calamita* (**g**) and *Bufotes viridis* (**h**) using species specific BamHI-800 sequences as a probe after two (**c**) or three (**a**,**e**,**g**,**h**) rounds of amplification. DAPI-stained metaphase chromosomes (**b**,**d**,**f**) corresponding to (**a**,**c**,**e**), respectively. Scale bar equivalent to 2.5 µm.

Due to limitations in sample availability, the chromosomal distribution of BamHI-800 repetitive DNA could only be analysed in four bufonid species: *Bufo bufo* (Italy), *Bufo spinosus* (Spain), *E. calamita* (Spain) and *Bufotes viridis* (Greece). The karyotypes of these species have 2n = 22, with six pairs of large and five pairs of small metacentric or submetacentric chromosomes. In situ hybridization using a species-specific BamHI-800 probe produced positive signals located at species-specific positions (Fig. 4).

*Bufo bufo* samples display intense positive hybridization signals on the centromere and short arm of the smallest pair of the karyotype (chromosome 11). Other faint signals are also observed on pericentromeric regions of all large chromosomal pairs, on the long arm of the 6th chromosomal pair, next to the secondary constriction corresponding to the NOR, and on the long arm of chromosome pair 7, close to the centromere (Fig. 4a,b).

**Figure 5:**
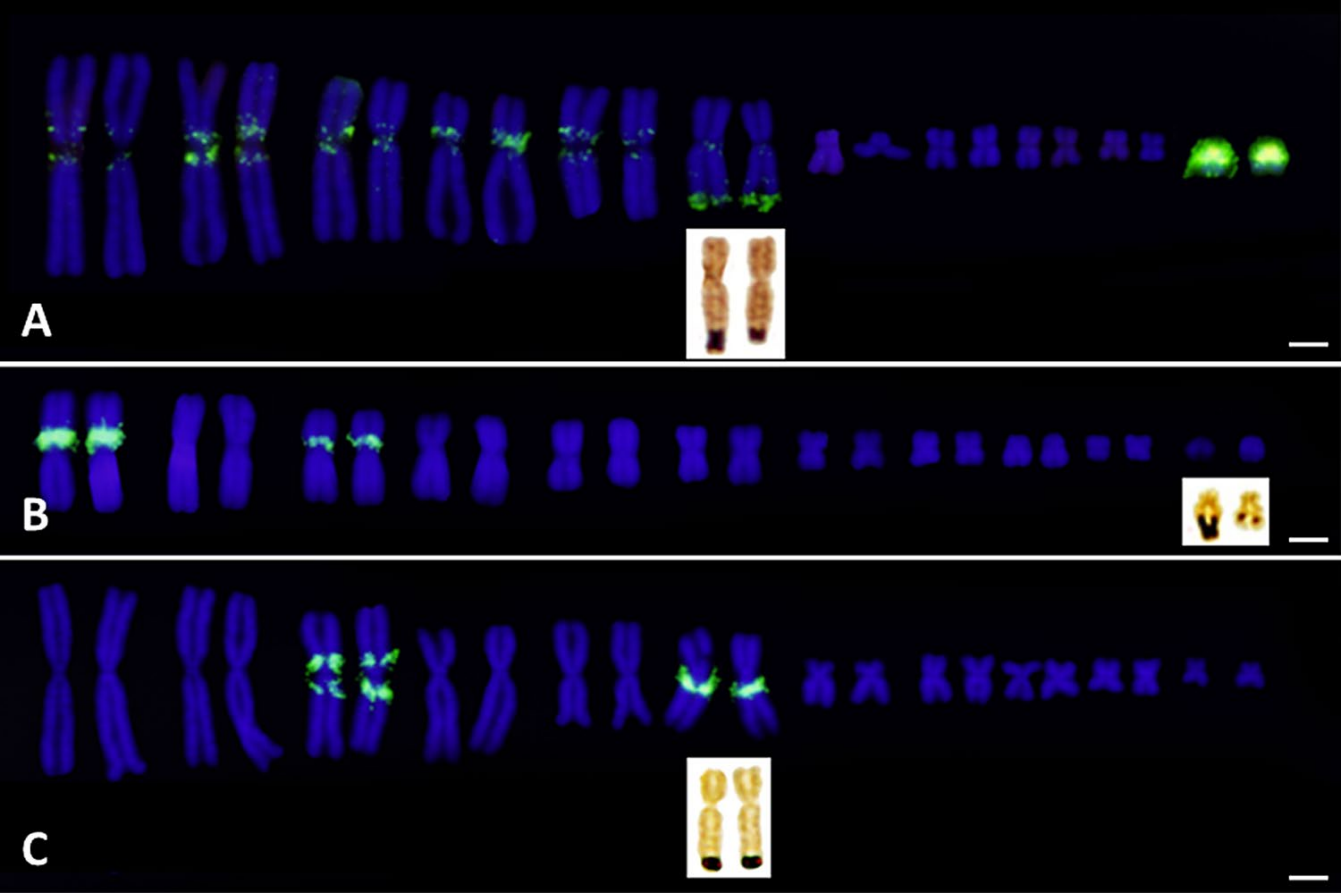
Karyotypes of *Bufo spinosus* (**a**), *Epidalea calamita* (**b**) and *Bufotes viridis* (**c**) hybridized with species-specific BamHI-800 sequences. The position of the NOR is indicated with an inset showing the corresponding Ag-stained chromosome pair. Scale bar equivalent to 2.5 µm.

The hybridization pattern in *Bufo spinosus* is similar to *Bufo bufo*, except for the absence of a positive signal on chromosome 7 (Fig. 4c,d). The signals are more evident after three rounds of immunological amplification (Figs. 4e,f and 5a). The presence and the intensity of the signal on the long arm of chromosome pairs 2 and 5 (close to the telomere and to the centromere, respectively) showed variability among samples. This variability suggests the existence of polymorphisms in the amount of BamHI-800 repetitive DNA in these chromosomal locations. Analysis of more samples from different geographic locations should be performed to check this hypothesis.

The positive signals in *E. calamita* sit on the short arm of two of the largest chromosome pairs (chromosomes 1 and 3). In both pairs, BamHI-800 is located close to the centromere, with the signal of pair 1 being more intense than that of pair 3 (Figs. 4g and 5b).

In *Bufotes viridis* BamHI-800 is located on the 3rd chromosomal pair, in pericentromeric position at both sides of the centromere, with the most intense signal on the short arm. In addition, there is a positive signal in the chromosomal pair that carries the NOR (pair 6), located on the long arm and close to the centromere (Figs. 4h and 5c). As opposed to *Bufo bufo*, in *Bufotes viridis* the signal is not located near to the NOR, but in the pericentromeric region of the same chromosome arm.

### Information about expression of BamHI-800

To check if this satellite DNA is transcribed, we searched for BamHI-800 sequences in published transcriptomes from several Bufonidae species. We did not detect expression of BamHI-800 sequences in assembled transcriptomes^23^. However, BamHI-800 sequences produce hits in BLASTN searches against Sequence Read Archive (SRA) data from several Bufonidae RNA sources. The number of hits obtained in several conditions (tissues, stage, spots, etc.) is shown in Supplementary Table S8.

## Discussion

We have characterized a new family of repetitive DNA (BamHI-800), isolated from *Bufo bufo* genomic DNA digested with BamHI. It is organized in tandem arrays of monomeric units of ca. 800 bp. Compared to other satellite DNAs in other amphibians, such as pBv PstI in *Bufotes viridis*^19^, Dp-sat1 in *Discoglossus pictus*^24^, PcP190 in Hyloidea^21^, or S1 in *Rana* ssp.^12,25–27^, BamHI-800 consists of a relatively large repetitive unit. Monomers of such length have not been identified when amphibian repetitive elements are characterized from next-generation sequence reads, e.g. in *Proceratophrys boiei*^16^.

Southern-Blot hybridization shows positive signals only in Bufonidae. BamHI-800 sequences were neither identified by Southern-Blot nor by specific PCRs in three other anuran families (e.g. Alytidae (*D. galganoi*), Pipidae (*X. tropicalis*), and Ranidae (*P. perezi*)). Fourteen bufonid toad species (*Bufo bufo*, *Bufo spinosus*, *Ba. brongersmai*, *Bufotes boulengeri*, *Bufotes luristanicus*, *Bufotes surdus*, *Bufotes viridis*, *Bufotes latastii*, *Bufotes balearicus*, *Bufotes siculus*, *D. melanostictus*, *E. calamita*, *R. marina*, and *S. arabica*) from seven genera exhibited similar banding patterns by Southern-Blot or PCR for BamHI-800. In addition, BLASTN searches in genome assemblies of Bufonidae confirmed BamHI-800 in *Bufo bufo* and *R. marina*, and provided new information in *Bufo gargarizans*. Genomes of other amphibian species showed no BLASTN hits (Supplementary Table S3), pointing to BamHI-800 as a Bufonidae-specific satellite DNA. Further analysis of closer outgroups (Hyloidea) and early diverging bufonid lineages are needed to confirm the distribution of this satellite DNA family.

Repetitive DNAs have been studied by PCR and sequencing, e.g. the S1 satellite of some species of *Rana*^12,26,27^, or the PcP190 satellite in Hyloidea^21^. In our study, the variability of BamHI-800 sequences obtained by traditional digestion-cloning is similar to that obtained by sequenced PCR products. Therefore, PCR does not select particular sequence groups from BamHI-800 sequences, but amplifies a wide range of variants of this repetitive DNA.

The BamHI-800 satellite-DNA family is conserved in the genomes of all studied bufonids. Genetic distances and phylogenetic groups suggest ten BamHI-800 subfamilies distributed in 2 variants. For some species, like *Bufo gargarizans*, *Ba. brongersmai*, *D. melanosticus*, *E. calamita*, *R. marina*, and *S. arabica*, the BamHI-800 V1-variant shows lower intraspecific variation than interspecific divergence, a sign of concerted evolution. This particular mode of evolution is the consequence of a two-level process called *molecular drive*, consisting of sequence homogenization and fixation^28,29^. Thus, high sequence homogeneity within a subfamily of satellite DNA monomers is a result of non-independent evolution of monomers, driven by non-reciprocal sequence transfer, such as unequal crossover^29^, gene conversion^30–32^, rolling circle replication^26,33,34^, transposition^35^ and may be others^2^. While homogenization depends on mechanisms of genomic turnover, fixation results from random chromosome assortment during sexual reproduction through meiosis and chromosome segregation. As a consequence, fixation depends on population factors. The outcome of this process is higher homogeneity of satellite monomers within lineages than between them. Thus, satellite DNA can evolve by a gradual accumulation of mutations, which results in the divergence of satellite sequences of reproductively isolated organismal groups^2^. Accordingly, it can be phylogenetically informative, for example, in species^36^, ecotype-specific variants, or phylogeographic clades^37^.

We did not find significant sequence divergence between different populations of the *Bufo bufo* group*.* This is interesting since we have compared BamHI-800 obtained from *Bufo bufo* sensu lato. The current taxonomy of the *Bufo bufo* species group distinguishes four species^38,39^: *Bufo bufo* (Linnaeus, 1758) from most of Europe (from northern and eastern France into Russia, including toads from Great Britain, Scandinavia, Italy, the Balkans, and the larger part of Turkey), *Bufo eichwaldi*^40^ from the Talysh mountains of Azerbaijan and Iran, *Bufo spinosus* (Daudin, 1803) from North Africa, Iberia and much of France, and *Bufo verrucosissimus* (Pallas, 1814) from the Caucasus and the north-eastern part of Turkey. According to their geographic distribution^41,42^, and their 16S sequence, our samples from Spain and Morocco correspond to *Bufo spinosus*, while samples from NCBI (London) and Bologna to *Bufo bufo*. However, in our maximum-likelihood analysis, the BamHI-800 sequences were not clustered according to geographical origin/species. Further studies with additional samples are required to uncover species- or population-specific differences.

Contrary to the situation in *Bufo bufo* species group, BamHI-800 V1-type sequences in *R. marina* have diagnostic positions that differentiate two Australian populations (Brisbane, Queensland (our sample) and Oombulgurri, Western Australia (Biosample: SAMEA104558286)). This suggests satellite diversification appears not always indicative of the divergence time. Considering that Australian *R. marina* can be traced back to 102 individuals introduced in 1935, the rapid BamHI-800 diversification might be explained by an initially very low population size followed by enormous population growth. Future analysis of BamHI-800 in *R. mari*na in its native S-American range could provide information on the effect of biological conditions on the evolution of satellite DNAs.

BamHI-800 sequences in *Bufotes* could not be clearly classified into species-specific subfamilies. In this genus, intraspecific variability is greater than interspecific divergence, and consequently, BamHI-800 sequences appear scattered in the phylogenetic tree. However, two differentiated groups were identified in *Bufotes*. These groups correspond with the two ancient clades of diploid toads: taxa diversified around the Mediterranean Basin and the Middle East and diploid taxa occurring between Iran, Iraq, Pakistan, and the Indian Himalayas^43–45^.

*Barbarophyne brongersmai* previously occupied a controversial taxonomic position. It was initially placed in the “*Bufotes viridis*–*E. calamita* group” based on morphology^46^, and then into the “*Bufotes viridis* group”^47^. Subsequent research on larval morphology, karyology, osteology, and bioacoustics revealed that *Ba. brongersmai* is well-diverged from all other Western Palearctic bufonid taxa^48–50^. Later mitochondrial and nuclear DNA-based phylogenies^43,51–53^ distinguished and clearly separated *Ba. brongersmai* from green toads (*Bufotes*), which was confirmed by^54^. According to this phylogenetic position, it is not surprising that the BamHI-800 sequences of *Ba. brongersmai* appear in an independent group in the phylogenetic tree.

The BamHI-800 sequences found in two other bufonid genera, *R. marina* and *D. melanostictus*, are of two types: those clustering in separate subfamilies as V2 variants and those phylogenetically related to the V1-variant of BamHI-800. The ‘library hypothesis’, not mutually exclusive with concerted evolution, assumes the existence of a collection (‘library’) of satellite DNAs shared by a species group. Accordingly, these taxa share a library of distinct conserved satellite DNA monomers^4,55,56^ or subfamilies, differentially amplified in each taxon. Variability between sequences reduces the homogenization effects of molecular mechanisms of non-reciprocal exchange, and sequence variants persist as a library^57–59^. Any of these variants may be differentially amplified in each taxon. Thus, in different lineages (species or genera), the subsequent replacement or amplification of one sequence variant can take place.

Replacement of a sequence variant by another in different species is a common feature of satellite DNAs^2,60^. When it occurs, unrelated species-specific dominant repeats reveal the presence of low-copy counterparts of each in others. This has been suggested in insects^55–57^ and plants^61,62^, and could explain our observations in Bufonidae, in which both hypotheses (concerted evolution and differential amplification of satellite variants) appear applicable.

FISH-localization of BamHI-800 in four species (*Bufo bufo*, *Bufo spinosus*, *Bufotes viridis*, and *E. calamita*) is not conserved but shows species-specificity in different chromosomes. BamHI-800 is mostly a component of the repetitive DNA in most pericentromeric regions of *Bufo bufo* species group (mainly in the large chromosomes and in the smallest pair). However, our comparison of FISH and C-banded karyotypes shows this is not the case in the other two species. Some C-positive bands could present BamHI-800, e.g. two pericentromeric C-bands on the short arm of pairs 1 and 3 in *E. calamita*^15^, occurring in the same position as BamHI-800. Similarly, in *Bufotes viridis*, the pericentromeric C-band on the short arm of chromosome 3 and on the long arm of chromosome 6 may probably show BamHI-800 accumulation. The short arms of chromosome 3 in *Bufotes viridis* also exhibit pBv satellites^19^, apparently at the same location as BamHI-800. Our analyses show no relation between these two repetitive DNA families.

Of note is the association between the NOR and the accumulation of BamHI-800 in *Bufo bufo* and *Bufo spinosus*. Considering that the NOR in *Bufo bufo* is flanked by constitutive heterochromatin^15^, the telomeric BamHI-800 FISH-signal of pair 6 may correspond to one of the C-positive bands flanking the NOR. BamHI-800 sequences in *Bufo viridis*, and *E. calamita* are not associated with NORs, although this repetitive DNA in *Bufotes viridis* sits on the NOR-bearing chromosome 6^63^. The NOR regions of *Bufo bufo*/*Bufo spinosus* and *Bufo viridis* are located in subtelomeric and telomeric positions of the long arm of the sixth chromosome pair, while they are located at terminal position of the long arm of the eleventh chromosomal pair in *E. calamita*^15^.

BamHI-800 is associated with constitutive heterochromatin of centromeric/pericentromeric regions. Considering its location, a role of these sequences in centromere function/heterochromatin maintenance could be proposed. The existence of hits for this repetitive DNA in adult transcriptomes from several Bufonidae species is of interest. More studies about its expression in more species and earlier developmental stages could add more information to this interesting question regarding a monomer unit with a long size.

## Conclusions

This work provides new data on evolutionary dynamics of satellite DNA in amphibians. This is the first study to reveal the occurrence of a specific satellite DNA family (BamHI-800) and its evolution in bufonid toads. Comparative analysis of BamHI-800 in seven bufonid toad genera shows that the structure, organization, and chromosomal distribution of this satellite DNAs is a relevant feature to distinguish the genomes of related species. The consequences of concerted evolution on intra-species homogenization depend on the species analysed. Most species clearly show specific differences (in *R. marina* even differentiated between populations), while in others the homogenization process is still ongoing. Moreover, the existence of variants in some species supports the ‘library hypothesis’. The results indicate that this repetitive DNA has a high degree of evolutionary conservation in one of the largest anuran families.

## Methods

### Samples

Animals were euthanized by immersion in buffered 2% Tricaine methanesulfonate (MS 222; Sigma-Aldrich, Germany) and then decapitated and dissected. Samples from 14 amphibian species belonging to seven genera of Bufonidae have been analysed. Three other anurans (*Discoglossus galganoi*, *Xenopus tropicalis*, and *Pelophylax perezi*) were used as outgroups. The examined species, the number of individual, their origin, chromosome number and the number of sequences considered are listed in Table 1. Individuals from *Bufo bufo* species group analysed in this work were assigned to *Bufo bufo* or *Bufo spinosus* species according to the 16S sequence^64^.

This study was approved by the relevant Institutional Animal Care and Use Committee (Bioethics Committee of the University of Jaén, 2009). Samples were collected in accordance with regulations for the protection of terrestrial wild animals. Capture permits for *Bufo bufo*, *Bufo spinosus E. calamita*, *D. galganoi*, and *P. perezi* were provided by the Junta de Andalucía, Dirección General de Gestión del Medio Natural (2010, 2012). Sampling green toad clutches in Crete was done under permit 115790/229. Care and sacrifice of animals used in this research was conducted in accordance with policies on experimental animals provided by Spanish and EU regulations. When applicable, this study is reported according to the ARRIVE guidelines.

### DNA extraction, repeat DNA isolation and cloning

Genomic DNA was extracted from skin or liver following the standard phenol–chloroform protocol^65^. Repetitive sequences from *Bufo bufo* and *E. calamita* were revealed by digestion of genomic DNA with several restriction endonucleases (EcoRI, HindIII, PstI, BamHI, AluI, SalI, KpnI y SmaI) according to the manufacturers’ protocols and subsequent agarose gel electrophoresis. After BamHI digestion, the intense bands of about 800 bp and 1600 bp were eluted (GeneJET Gel Extraction Kit, Thermo Fisher Scientific (Waltham, Massachusetts)) and cloned into BamHI-digested pUC19 vector (Fermentas (Vilnius, Lithuania)). Competent JM109 bacteria were transformed by thermal shock. Recombinant clones were denaturalized, dot-blotted onto nylon membranes and hybridized using the BamHI-800 digoxigenin-labelled band as a probe. Positive clones were sequenced using ABI BigDye Terminator kit (Applied Biosystems (Massachusetts, USA)) and T7 or SP6 universal primers. Nucleotide sequences were obtained in an ABI PRISM 3730xl capillary DNA analyser at the DNA sequencing Service from the Centro Nacional de Investigaciones Oncológicas (CNIO (Madrid, Spain)).

### Design of specific primers and PCR cloning

The sequences obtained from cloned BamHI-800 bands from the species *Bufo bufo* and *E. calamita* were used to design specific primers to PCR-amplify the repetitive sequences in other amphibian species (Supplementary Fig. S1). BamHI-800 sequences were PCR-amplified in other amphibian species in volumes of 50 μl, using 100 ng of genomic DNA, 0.7 U of Taq DNA polymerase (Bioline GmbH, Luckenwalde, Germany), 0.4 μM of each primer, 100 μM of each dNTP and a final MgCl_2_ concentration of 2 mM in 1× PCR buffer. The thermal conditions used were as follows: 1 cycle of 95 °C for 4 min; 33 cycles of 92 °C for 30 s, 52 °C for 45 s, 72 °C for 45 s; and a final extension step of 5 min at 72 °C.

The PCR products from several species were separated by agarose electrophoresis purified with the GeneJET Gel Extraction Kit (Thermo Fisher Scientific (Waltham, Massachusetts)) and cloned into pGEM-T Easy Vector (Promega (Wisconsin, USA)) as described by the supplier. Positive recombinant colonies were selected and sequenced as indicated. All sequences used in this work were deposited on GenBank under accession numbers ON491183–ON491407.

### Southern-blot and probe hybridization

Samples of genomic DNA from different species were digested with BamHI. The resulting fragments were separated by electrophoresis on a 1% agarose gel (5 V/cm) and blotted onto Hybond N + nylon membranes (GE Healthcare (Chalfont St. Giles, UK)) as previously described^65,66^. Pre-hybridization was done at 68 °C for 3 h in absence of formamide; hybridization was performed overnight at 60–68 °C, using the BamHI-800 band, digoxigenin-labelled by random primers (the optimal hybridization temperatures in each case were obtained experimentally). Additionally, species-specific PCR-labelled probes with digoxigenin-11-dUTP (Roche, Mannheim, Germany) were obtained from positive clones for BamHI-800 sequences, using BamHI-800-F1/R1 as primers. The post-hybridization washes were done in agitation. The wash I (2 × 15 min each) was performed at room temperature in 2 × SSC/0.1% SDS, while the wash II (2 × 30 min each) was done at the hybridization temperature in 0.5 × SSC/0.1% SDS. Hybridization signals were detected with an anti-digoxigenin-11-dUTP antibody conjugated with alkaline phosphatase (Roche, Mannheim, Germany) and revealed using NBT-BCIP as substrate (Roche, Mannheim, Germany) according to the supplier’s recommendations.

### Sequence analysis

Multiple sequence alignments were performed with MUSCLE^67^ and edited manually in MEGA^68^. Sequence similarity searches were implemented at NCBI (http://Blatst.ncbi.nlm.nih.gov/Blatst.cgi), using blastn suite (megablast and discontiguous megablast) with default parameters^69^. Similarities with known repetitive elements were queried in Repbase^70^ and NCBI GenBank.

BamHI-800 sequences were searched in NCBI using BLASTN in standard databases (nr/nt) or in amphibian genome databases (Supplementary Table S3). The BLAST algorithm was optimized for more dissimilar sequences (discontiguous megablast), removing filtering and mask from algorithm parameters. Sequence from clone Bbuf.BH2_B-86 was used as entry query sequence in all cases except for *R. marina* genome, where sequence from clone Rmar_PCR1.1-5 was used. The hits obtained after BLASTN searches in *Bufo bufo*, *Bufo gargarizans* and *R. marina* genome databases were retrieved and complete sequences used in this analysis.

Dot Plot analysis was performed with YASS^71^ (https://bioinfo.lifl.fr/yass/yass.php), and DNA secondary structure prediction was modelled with RNA structure^72^ (V 6.4) with command line: Fold Predict1.fasta Fold.ct -loop 30 -maximum 20 -percent 10 -temperature 310.15 -window 3.

Evolutionary analyses were conducted in MEGA X^68^. The nucleotide substitution model with the lowest BIC (Bayesian Information Criterion) score was chosen (T92 + G): evolutionary divergence between sequences was estimated using the Tamura 3-parameter model^73^, and the rate of variation among sites was modelled with a gamma distribution. All ambiguous positions were removed for each sequence pair (pairwise deletion option). Standard error estimates were obtained by a bootstrap procedure (1000 replicates). Maximum likelihood (ML) analysis using the Tamura 3-model of sequence evolution^73^ was employed to generate a sequence tree. In our analysis, all positions with less than 95% site coverage were eliminated, and ambiguous bases were allowed at any position (partial deletion option). No big differences were observed when all positions with gaps are retained (all positions) or removed (complete deletion). The initial tree(s) for the heuristic search were obtained automatically by applying Neighbour-Joining and BioNJ algorithms to a matrix of pairwise distances estimated using the Maximum Composite Likelihood (MCL) approach, and then selecting the topology with the superior log likelihood. The significance of the phylogenetic lineages was assessed by bootstrap support values, calculated for 1000 replicates^74^.

Information about expression of BamHI-800 sequences was obtained using BLASTN optimized for more dissimilar sequences (discontiguous megablast) on NCBI Sequence Read Archive (SRA) data from Bufonidae RNA sequences. When available, the query sequence was from the same species/genus. In any other case, the sequence used was from clone Bbuf.BH2_B-86. Default algorithm parameters were used except for the lack of filtering and masking.

### Chromosome preparation and fluorescent in-situ hybridization (FISH)

Chromosome preparations were obtained from bone marrow cells of adult individuals or from cell culture from tadpoles according to the protocols described in^75^.

FISH was performed as previously described in ^76^. The probe used was a BamHI-800 positive clone from the same species as the chromosome preparations (Bbu-BamHI-800-86 in *Bufo bufo*, Bca-BamHI-800-9 and Bca-BamHI-800-10 in *E. calamita* and Bvi-BamHI-800-5 in *Bufotes viridis*), PCR-labelled with biotin-16-dUTP (Roche (Mannheim, Germany)). When indicated, three rounds of immunological amplification with avidin-FITC/anti-avidin–biotin were used. For each species and sample, more than 20 hybridized metaphase plates were photographed and analysed. The images were taken with FITC and DAPI filters in an Olympus BX-51 fluorescence microscope. Images were processed with Adobe Photoshop. Processing was applied equally across the entire image.

## Supplementary Information

Supplementary Figure S1.

Supplementary Figure S2.

Supplementary Figure S3.

Supplementary Figure S4.

Supplementary Figure S5.

Supplementary Figure S6.

Supplementary Figure S7.

Supplementary Table S1.

Supplementary Table S2.

Supplementary Table S3.

Supplementary Table S4.

Supplementary Table S5.

Supplementary Table S6.

Supplementary Table S7.

Supplementary Table S8.

Supplementary Table S9.

Supplementary Information.

## Data Availability

All BamHI-800 sequences obtained in this study were submitted to Genbank (Accession numbers: ON491183–ON491407). All data generated and analysed during this study are included in this published article and its Supplementary Information files.
