# Supplementary material for: Identification and characterization of a new family of long satellite DNA, specific of true toads (Anura, Amphibia, Bufonidae)
Source: Sci Rep. 2022 Aug 17;12:13960. doi: 10.1038/s41598-022-18051-9 (PMC9385698; doi:10.1038/s41598-022-18051-9)
Supplement: Supplementary file 1 — Supplementary Figure S1. [file 41598_2022_18051_MOESM1_ESM.pdf]

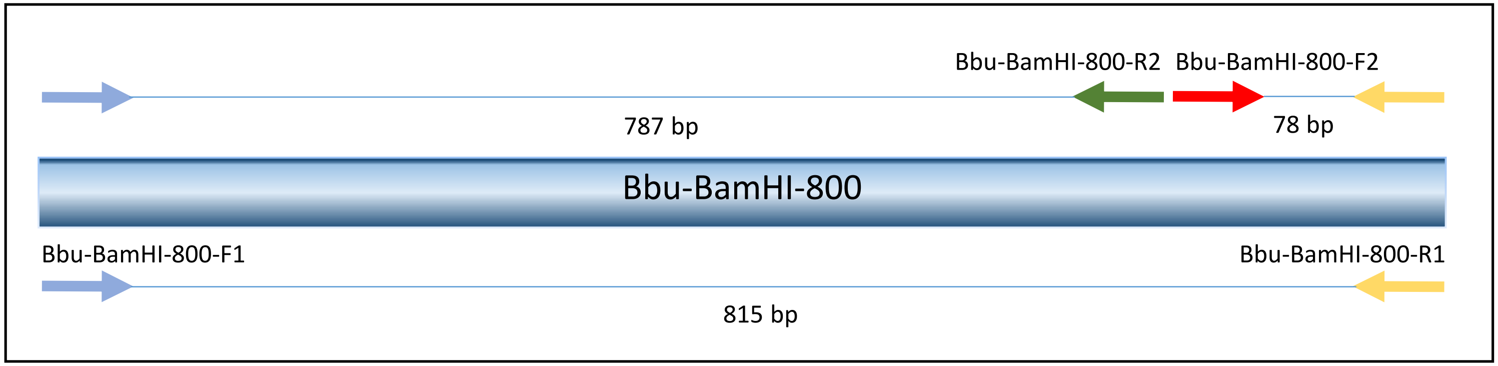

NAME	SEQUENCE 5'-3'	SIZE	Tm PrimerBlast	Tm IDT	%GC
Bbu-BamHI-800-F1	GGATCCATGCACATCAACAGGTATG	25	56.06	57.90	48.00
Bbu-BamHI-800-R1	GGATCCACACCTGACTGAAGAATG<br>CATTCTTCAGTCAGGTGTGGATCC	24	55.23	57.40	50.00
Bbu-BamHI-800-F2	GACATCACAATAGACCCTTGCTG	25	56.06	55.70	48.00
Bbu-BamHI-800-R2	ATCATCCATGGATGCTGAAGGG<br>CCCTTCAGCATCCATGGATGAT	22	54.23	57.10	50.00

Supplementary Figure S1: Primers used in this work.
