# Supplementary material for: Identification and characterization of a new family of long satellite DNA, specific of true toads (Anura, Amphibia, Bufonidae)
Source: Sci Rep. 2022 Aug 17;12:13960. doi: 10.1038/s41598-022-18051-9 (PMC9385698; doi:10.1038/s41598-022-18051-9)
Supplement: Supplementary file 2 — Supplementary Figure S2. [file 41598_2022_18051_MOESM2_ESM.pdf]

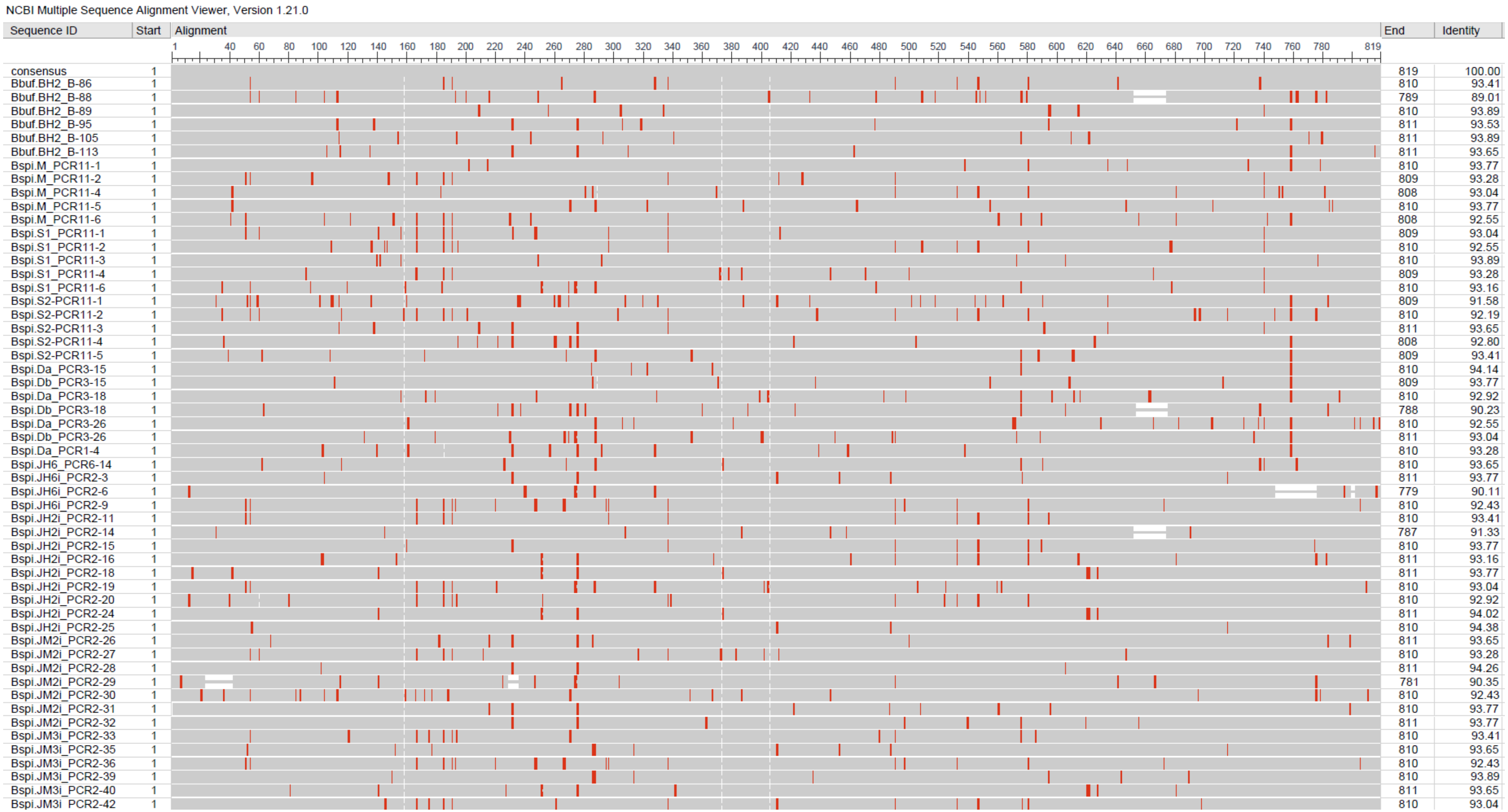

Supplementary Figure S2: Graphical display of multiple alignment of BamHI-800 from *B. bufo* species group (NCBI Multiple Sequence Alignment Viewer). Start: First position; End: number of bp of the sequence; Identity: % of identity with consensus sequence.
