# Supplementary material for: Identification and characterization of a new family of long satellite DNA, specific of true toads (Anura, Amphibia, Bufonidae)
Source: Sci Rep. 2022 Aug 17;12:13960. doi: 10.1038/s41598-022-18051-9 (PMC9385698; doi:10.1038/s41598-022-18051-9)
Supplement: Supplementary file 3 — Supplementary Figure S3. [file 41598_2022_18051_MOESM3_ESM.pdf]

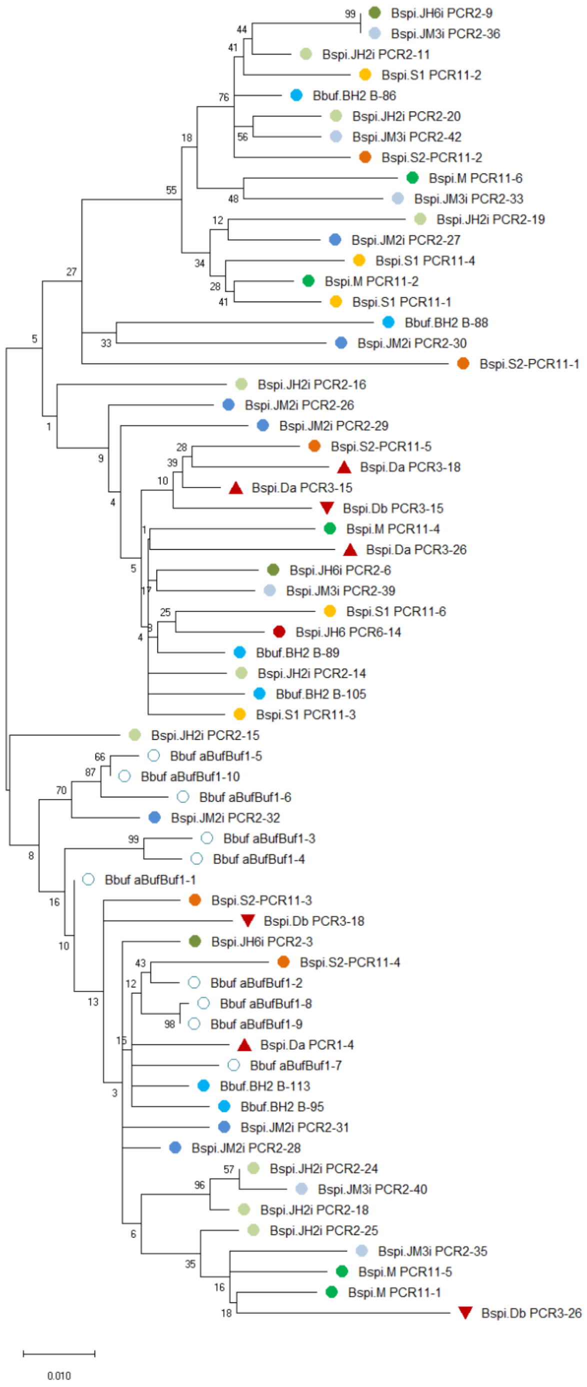

Supplementary Figure S3.
Maximum Likelihood tree for BamHI-800 from *Bufo bufo* species group using Tamura 3-model of sequence evolution [73]. A discrete Gamma distribution was used to model evolutionary rate differences among sites (5 categories (+*G*, parameter = 0.5128)). The tree with the highest log likelihood (-5562.92) is shown. The tree is drawn to scale, with branch lengths measuring the number of substitutions per site. The percentage of trees in which the associated taxa clustered together is shown next to the branches. This analysis involved 64 nucleotide sequences and 807 positions in the final dataset (all positions with less than 95% site coverage were eliminated, and ambiguous bases were allowed at any position (partial deletion option)). Sequences ID and symbols at branch leafs as in Supplementary Table S1.
