# Supplementary material for: Identification and characterization of a new family of long satellite DNA, specific of true toads (Anura, Amphibia, Bufonidae)
Source: Sci Rep. 2022 Aug 17;12:13960. doi: 10.1038/s41598-022-18051-9 (PMC9385698; doi:10.1038/s41598-022-18051-9)
Supplement: Supplementary file 4 — Supplementary Figure S4. [file 41598_2022_18051_MOESM4_ESM.pdf]

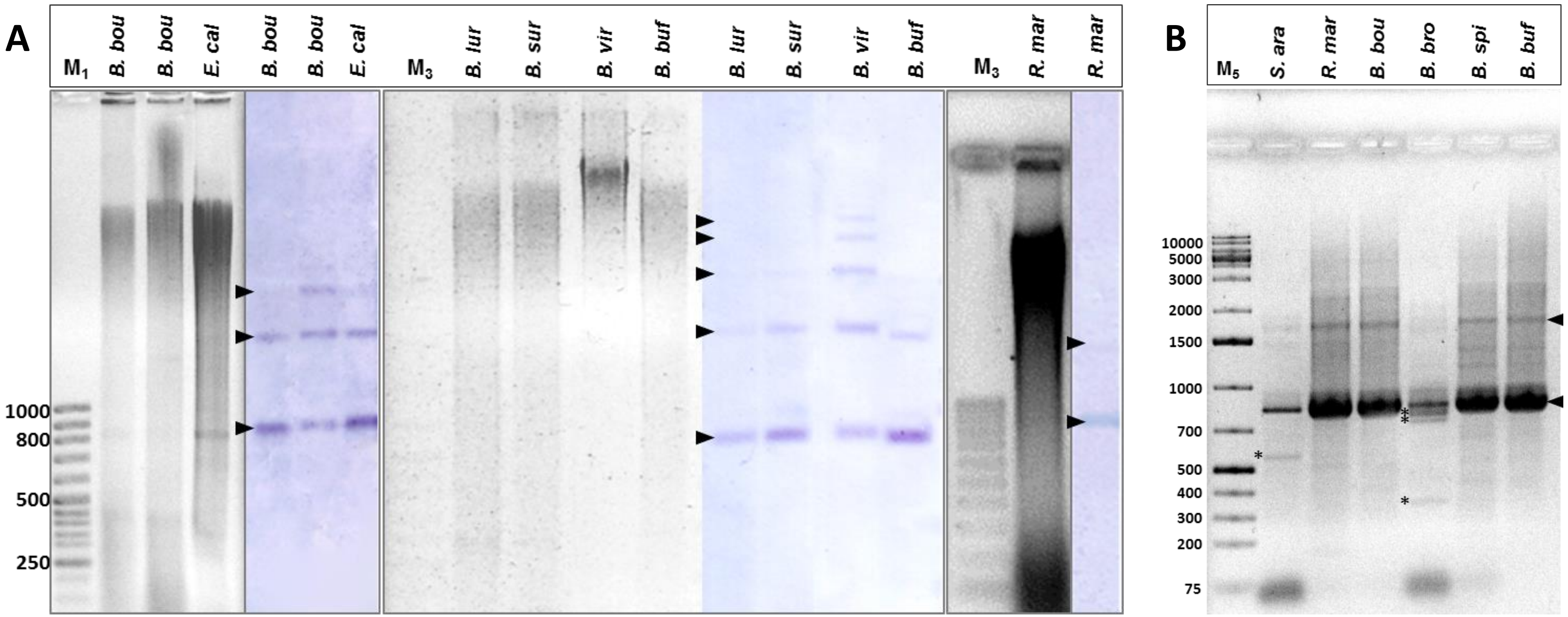

Supplementary Figure S4. a) Inverted image of agarose gel electrophoresis and corresponding Southern-blots of BamHI-digested genomic DNA of various Bufonidae species hybridized with BamHI-800 dig-labelled probe. *R. marina* and *Bufotes luristanicus* samples show lower signal intensity due to lower quality of genomic DNA. Comparative PCR with primer pair PCR1 in several Bufonidae species. **b**) PCR with primer pair PCR1 in species *S. arabica* (S. ara), *R. marina* (R. mar), *Bufotes boulengeri* (B. bou), *Ba. brongersmai* (B. bro), *Bufo spinosus* (B. spi) and *Bufo bufo* (B. buf). Arrow heads point to monomer and dimer bands. *S. arabica* and *Ba. brongersmai* produce less intense bands, together with some extra bands of lower size (*). The bands of 4000, 7000 and 20000 bp have not been pointed out in the molecular weight marker. For a detailed description of the molecular weight markers see Supplementary Table S9.
