# Supplementary material for: Identification and characterization of a new family of long satellite DNA, specific of true toads (Anura, Amphibia, Bufonidae)
Source: Sci Rep. 2022 Aug 17;12:13960. doi: 10.1038/s41598-022-18051-9 (PMC9385698; doi:10.1038/s41598-022-18051-9)
Supplement: Supplementary file 5 — Supplementary Figure S5. [file 41598_2022_18051_MOESM5_ESM.pdf]

Supplementary Figure S5 (pg. 1/3)

A

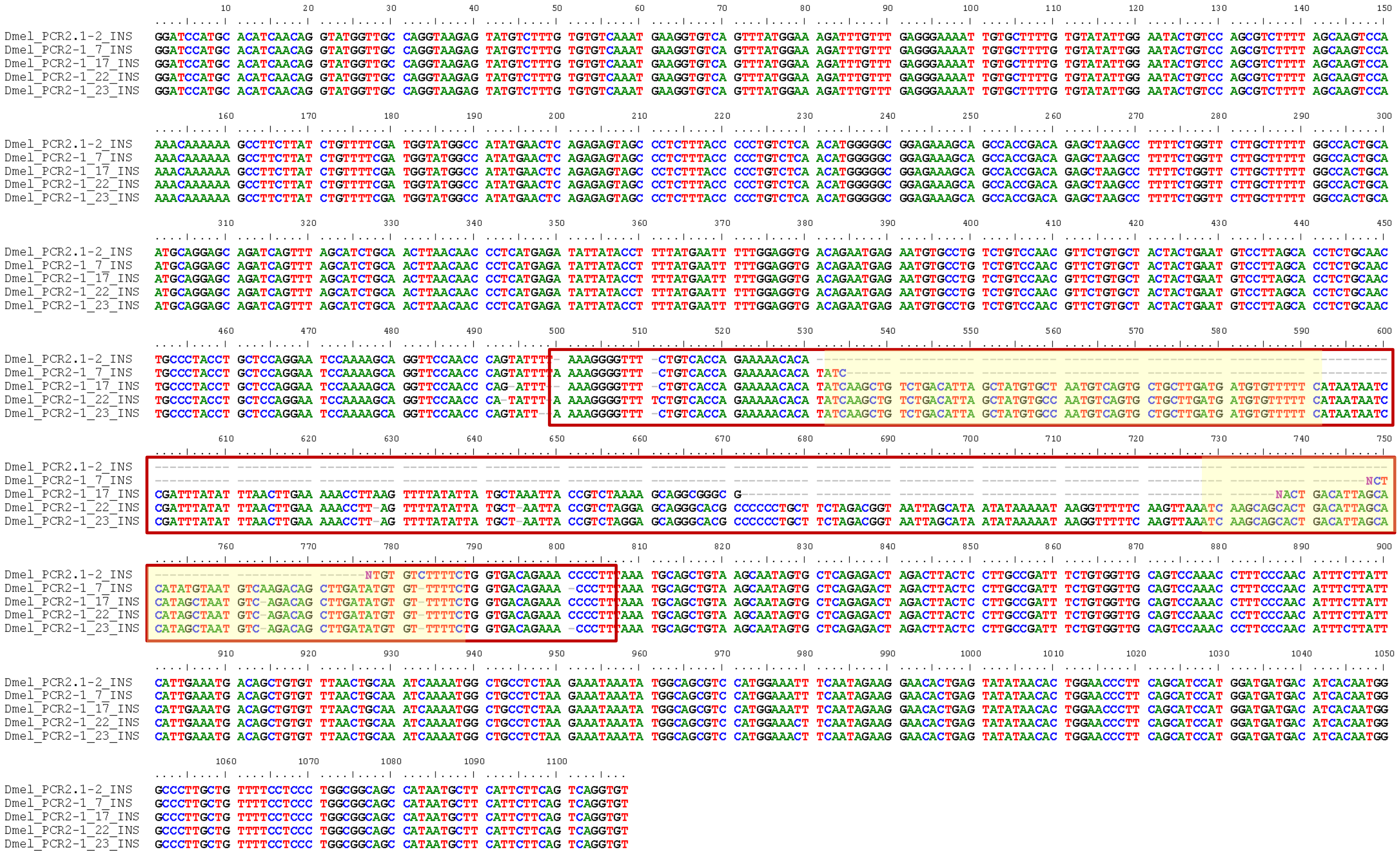

Supplementary Figure S5 (pg. 2/3)

B

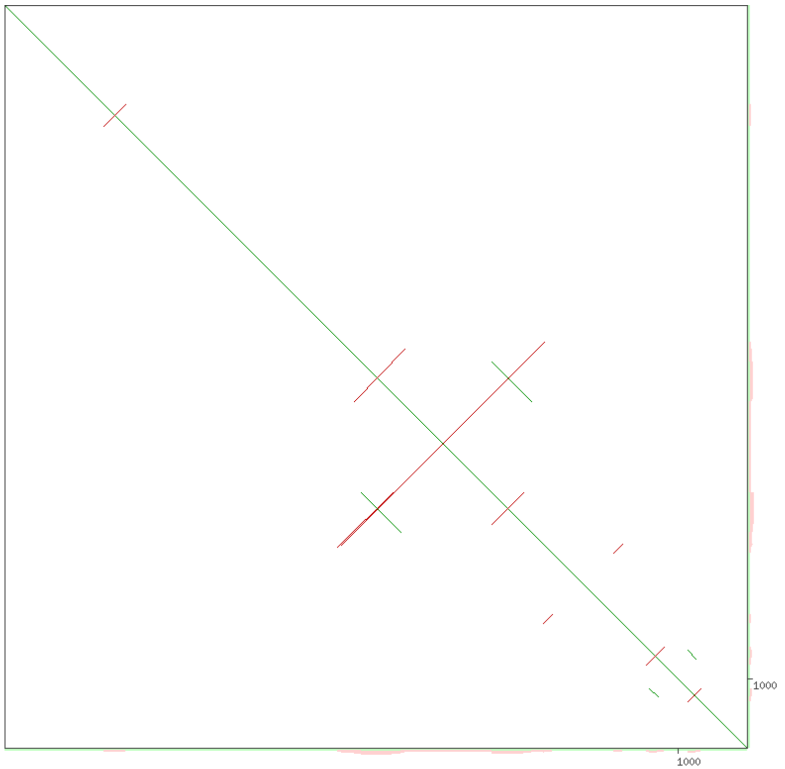


```
 |800      |790      |780      |770      |760      |750      |740      |730
AAAGGGGTTTCTGTCACCAGAAAAACACATATCAAGCTGTCTGACATTAGCTATGTGCTAATGTCAGTGCTGCTTGAT--
||:|||.|||||||||||||||||||||||||||||||||||||||||||||||||||:|||||||||||||||||||
AAGGGGTTTTCTGTCACCAGAAAAACACATATCAAGCTGTCTGACATTAGCTATGTGCCAATGTCAGTGCTGCTTGATGA
|500      |510      |520      |530      |540      |550      |560      |570

                                |720      |710      |700      |690      |680
-----------------------------TTAACTTGAAAAACCTTATTTTTATATTATGCTAATTACCGTCTAGAAGCA
                             ||||||||||||||||||.|||||||||||||||||||||||||||:||||
TGTGTTTTTCATAATAATCCGATTTATATTTAACTTGAAAAACCTTAGTTTTATATTATGCTAATTACCGTCTAGGAGCA
|580      |590      |600      |610      |620      |630      |640      |650

  |670      |660      |650      |640      |630      |620      |610      |600
GGGGGGCGTGCCCTGCTCCTAGACGGTAATTAGCATAATATAAAACTAAGGTTTTTCAAGTTAAATATAAATCGGATTAT
|||.:...:.|||||||:|||||||||||||||||||||||||||.||||||||||||||||||||
GGGCACGCCCCCCTGCTTCTAGACGGTAATTAGCATAATATAAAAATAAGGTTTTTCAAGTTAAAT--------------
|660      |670      |680      |690      |700      |710      |720

  |590      |580      |570      |560      |550      |540      |530      |520
TATGAAAAACACATCATCAAGCAGCACTGACATTGGCACATAGCTAATGTCAGACAGCTTGATATGTGTTTTTCTGGTGA
                 |||||||||||||||||:|||||||||||||||||||||||||||||||||||||||||||||
-----------------CAAGCAGCACTGACATTAGCACATAGCTAATGTCAGACAGCTTGATATGTGTTTTTCTGGTGA
                     |730      |740      |750      |760      |770      |780

  |510
CAGAAAACCCCTT
||||||.|||:||
CAGAAACCCCTTT
 |790
```

C

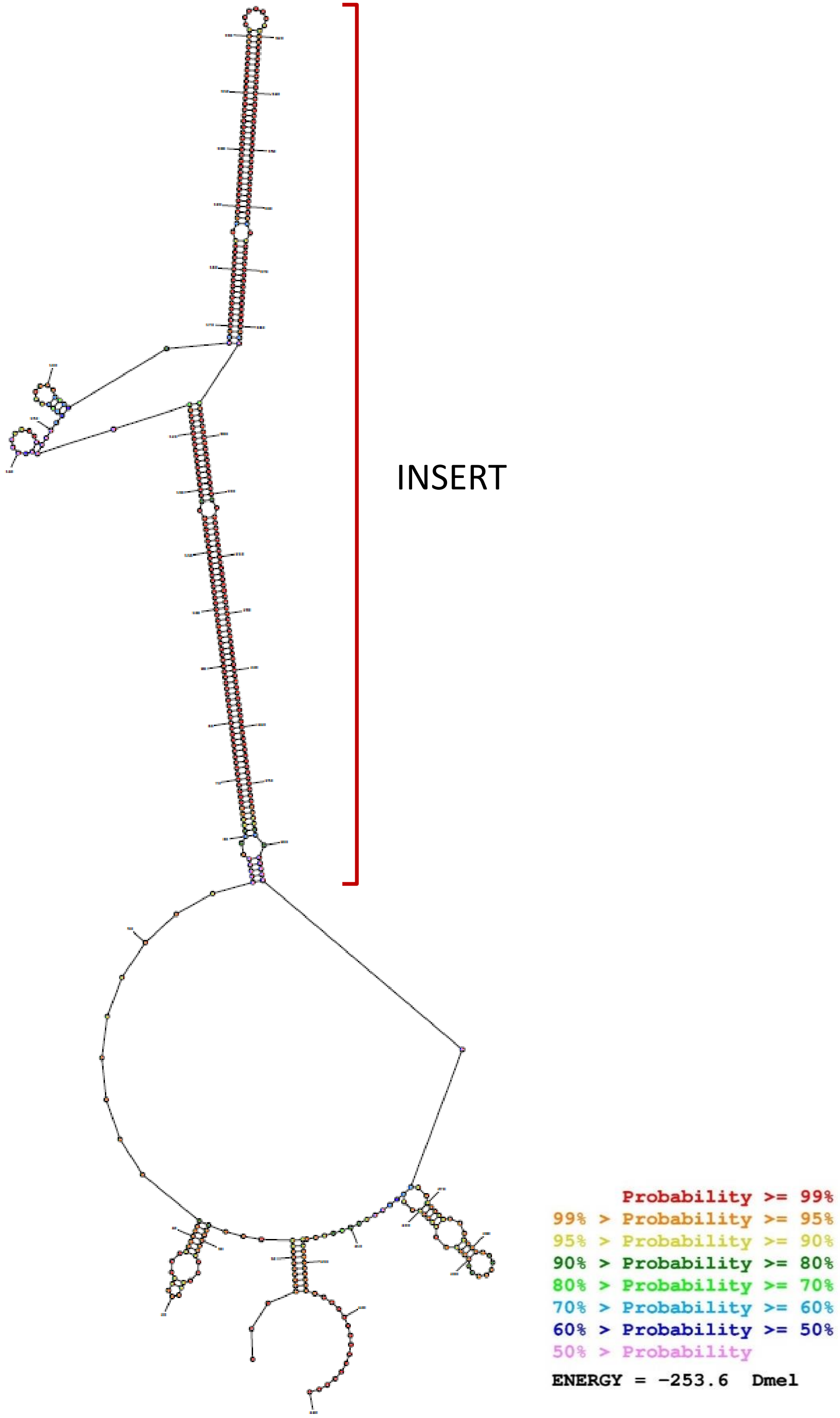

Supplementary Figure S5 (pg. 3/3)

D

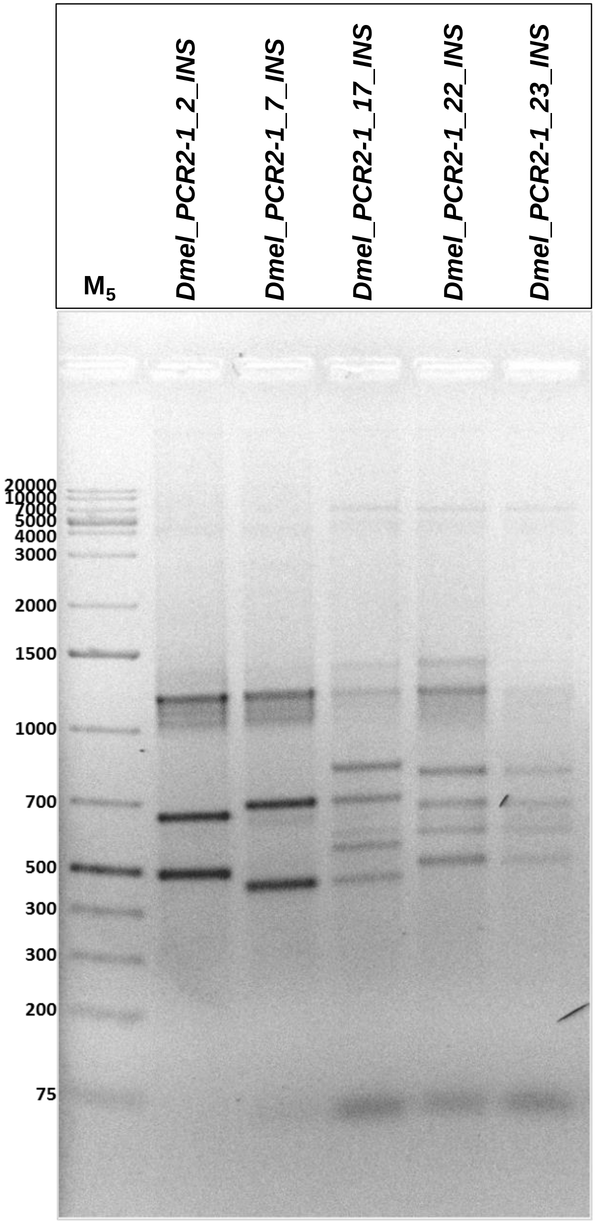

Supplementary Figure S5.
BamHI-800 in *D. melanosticus*. **a**. Alignment of the sequences of 5 clones from D. melanosticus (3 partial and 2 complete sequences). The N indicates the position where sequence loss quality. The inserted region is indicated as a red box. The beginning and end of the palindromic region is highlighted in yellow. **b**. Dot plot [71] of clone Dmel_PCR2-1_23_INS. The sequence of the longest palindromic region (longest red line) is included below. **c**. Secondary structure with lower free energy predicted [72] by clone Dmel_PCR2-1_23_INS. The long hairpin corresponds to the inserted region. **d**. PCR from clones of D. melanosticus with insert. When universal primers (M13F and M13R) are used, besides of the expected band of 1100 bp, several extra bands of lower size are observed. These artifacts are probably due to the presence of the palindromic regions identified, and may be responsible for the problems observed to sequence these clones. For a detailed description of the molecular weight marker see Supplementary Table S9.
