# Supplementary figures and images for: Identification and characterization of a new family of long satellite DNA, specific of true toads (Anura, Amphibia, Bufonidae)
Source: Sci Rep. 2022 Aug 17;12:13960. doi: 10.1038/s41598-022-18051-9 (PMC9385698; doi:10.1038/s41598-022-18051-9)

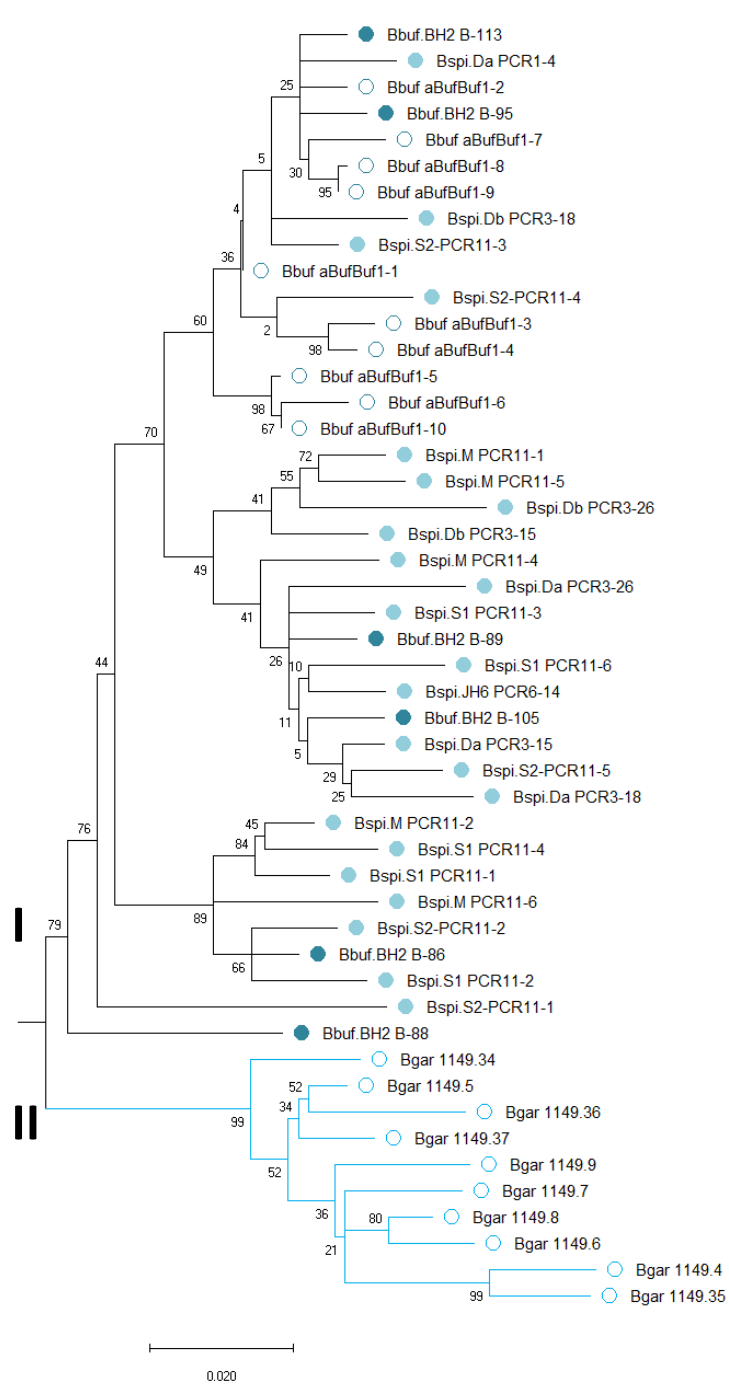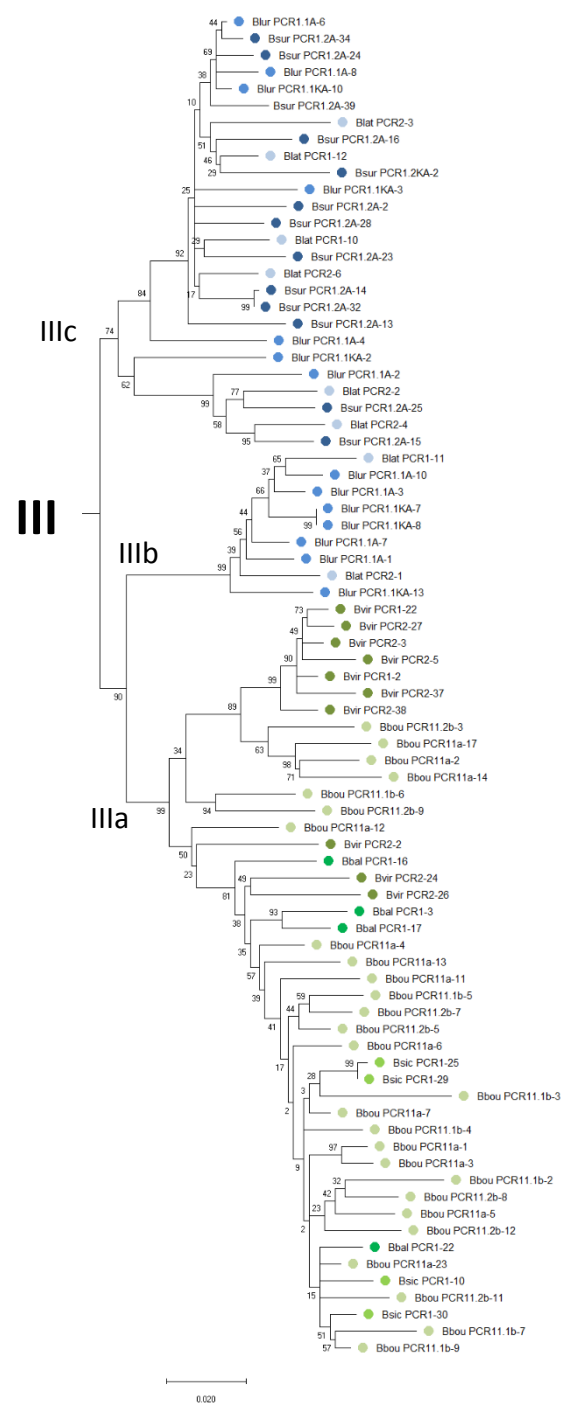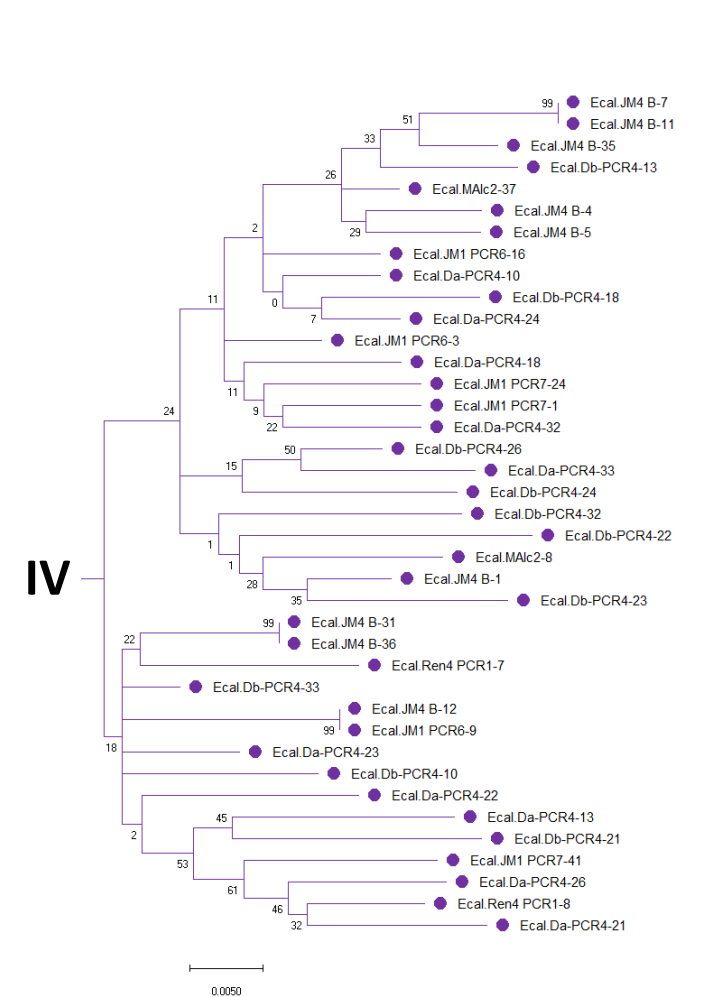

**Supplementary Figure S6: Subtrees from Figure 2.**  
Group ID is indicated according to Table 3.

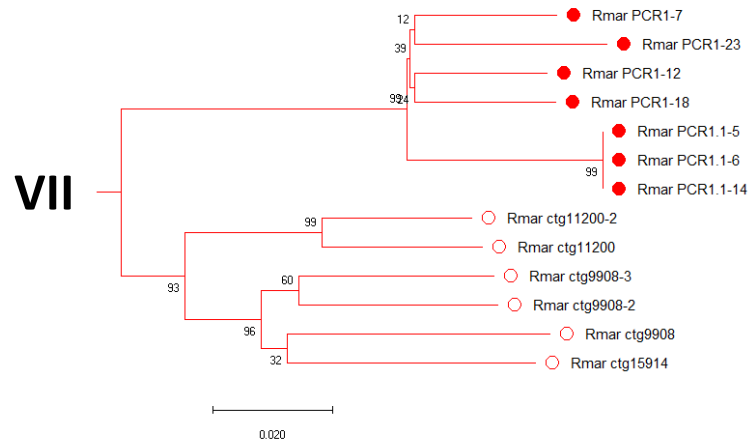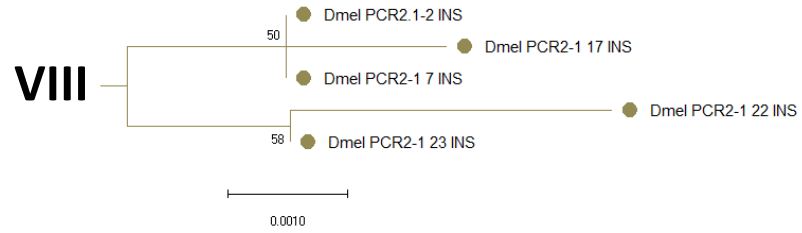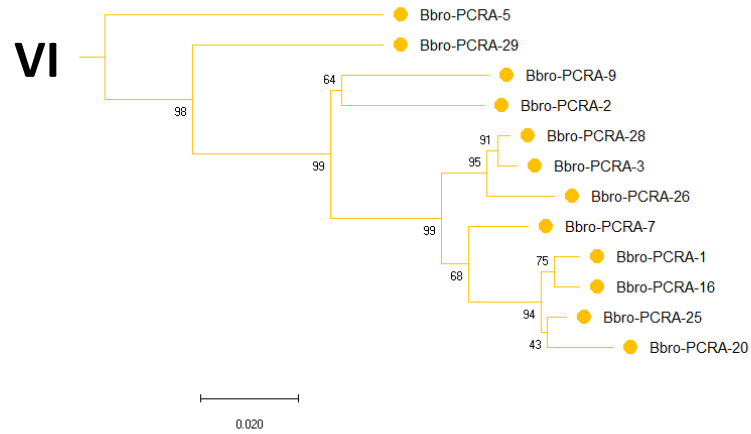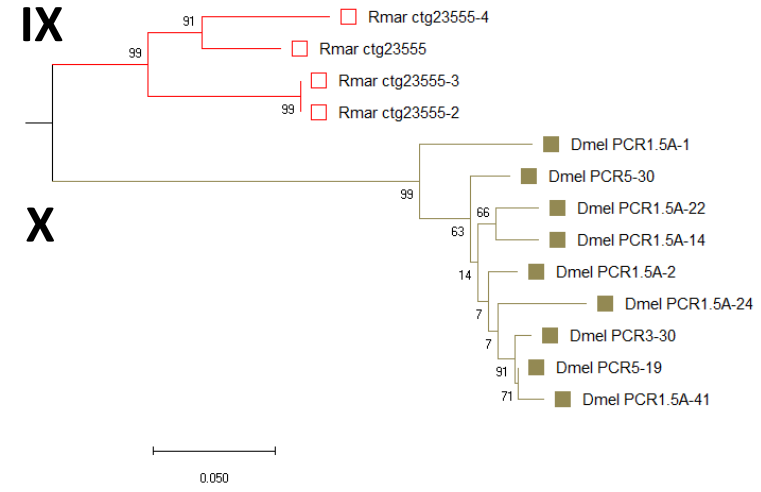

**X**

Supplement: Supplementary file 6 — Supplementary Figure S6. [file 41598_2022_18051_MOESM6_ESM.pdf]

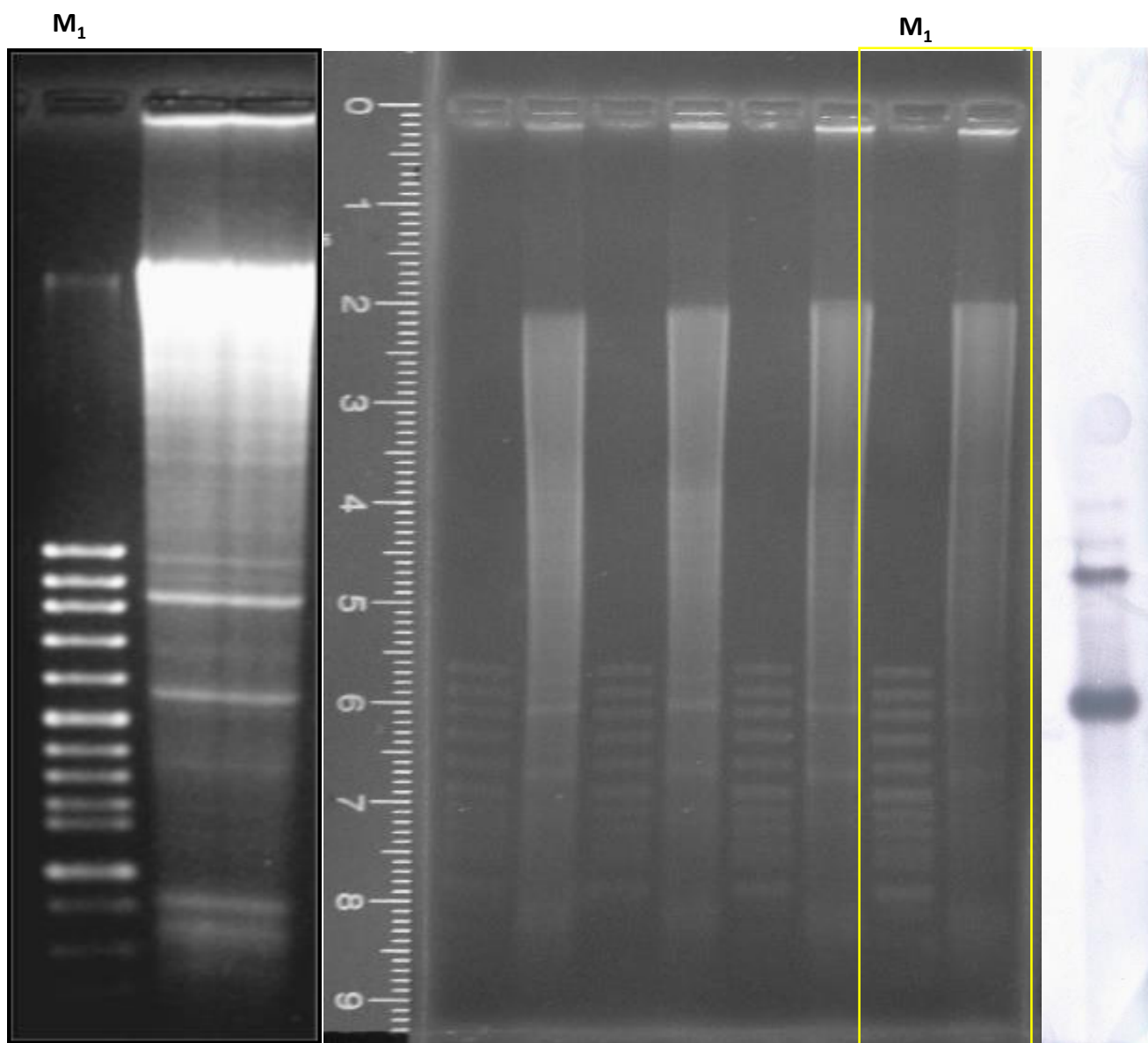

Fig. 1a

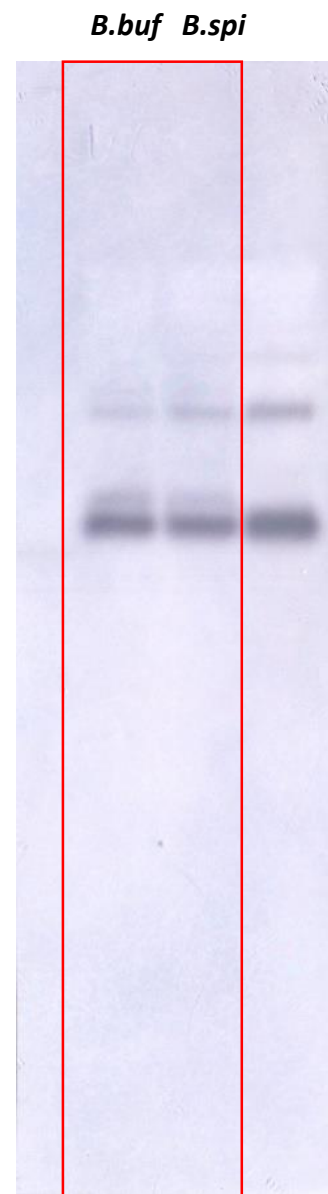

Fig. 1c

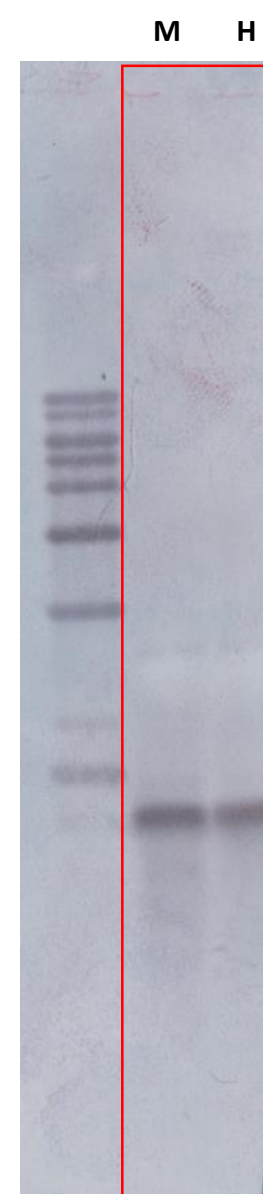

Fig. 1d

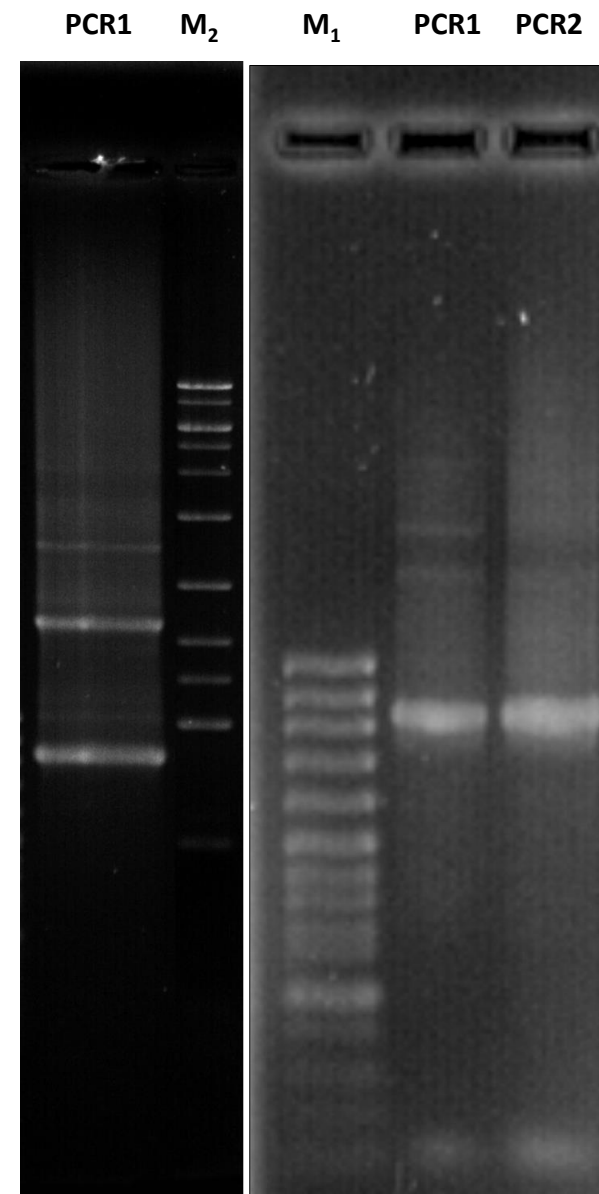

Fig. 1e

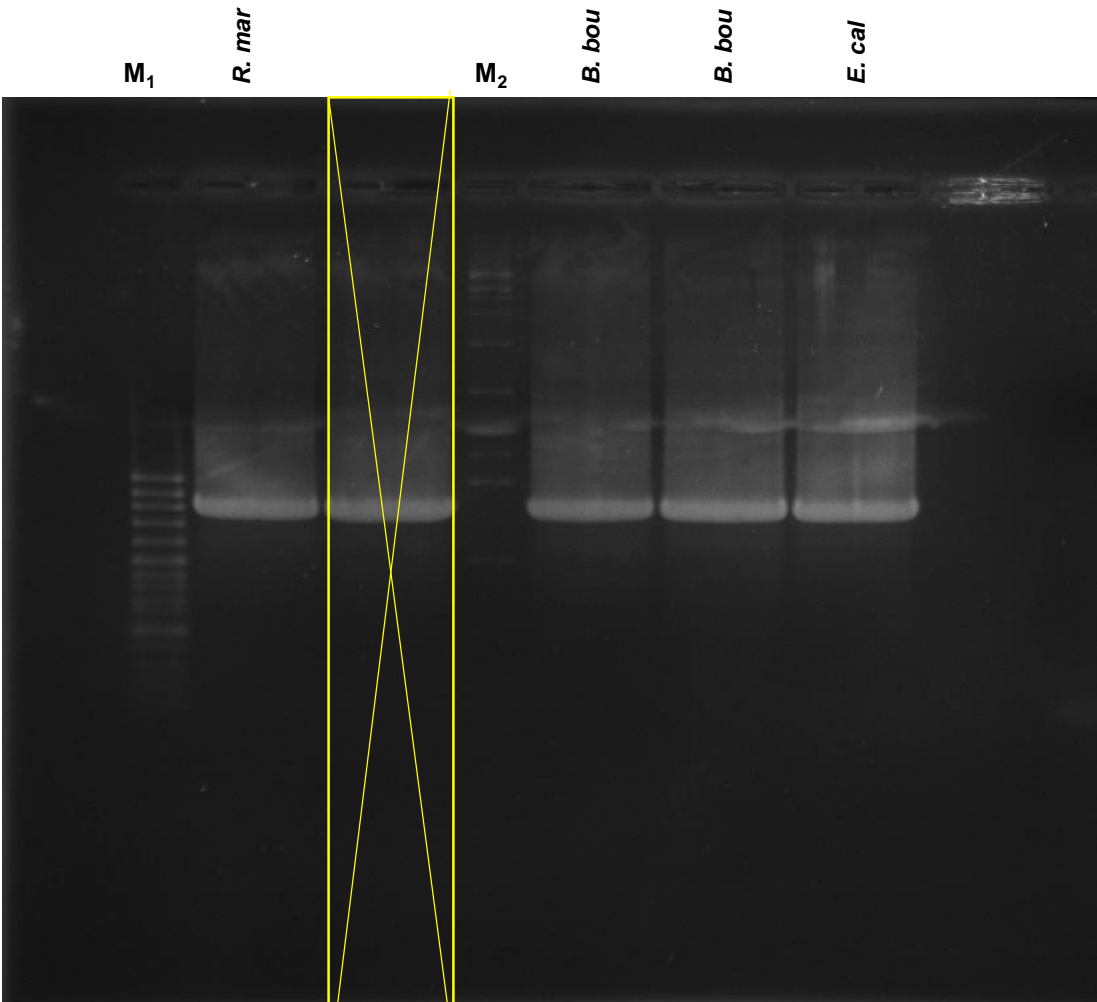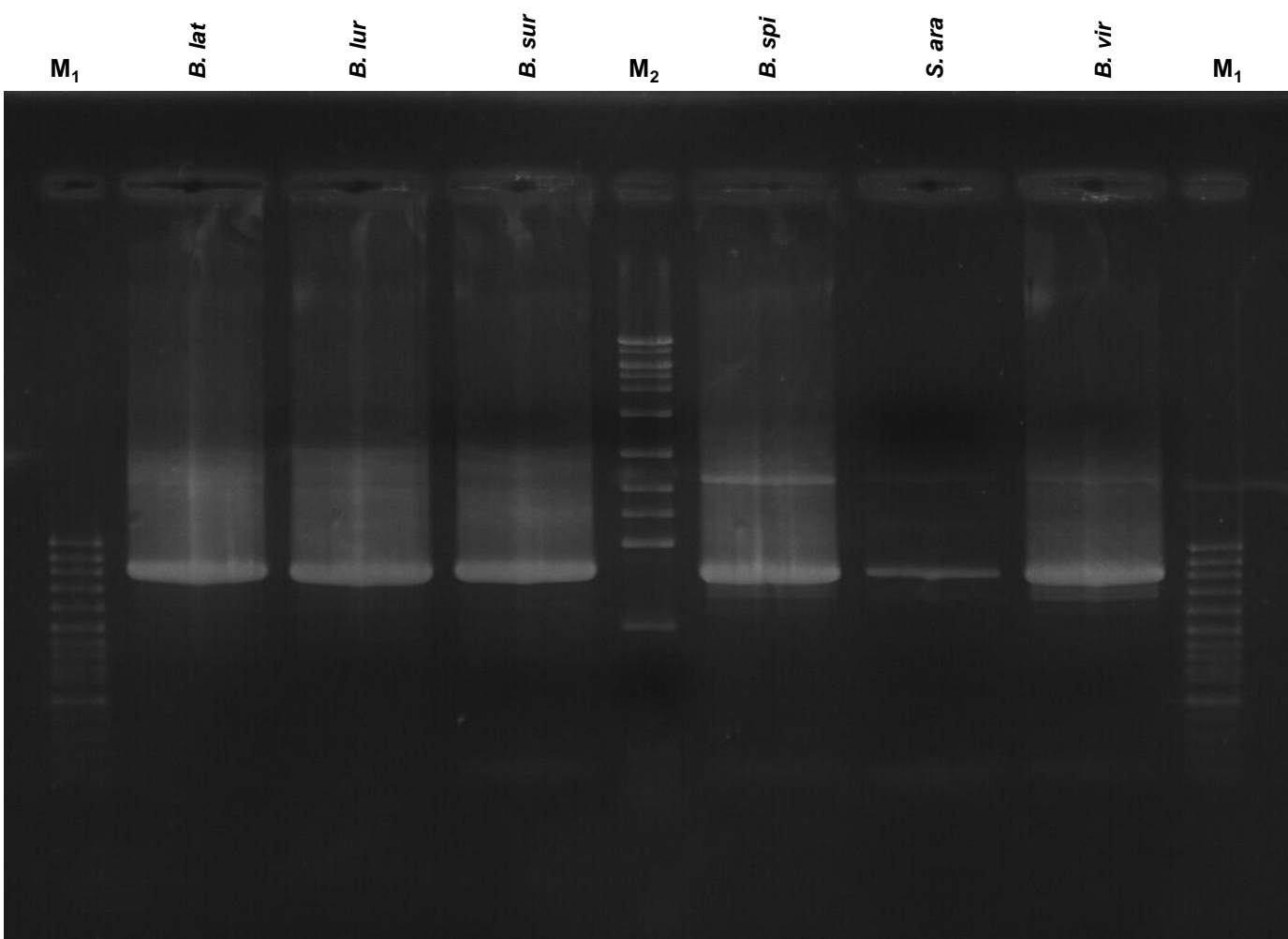

Fig. 1f

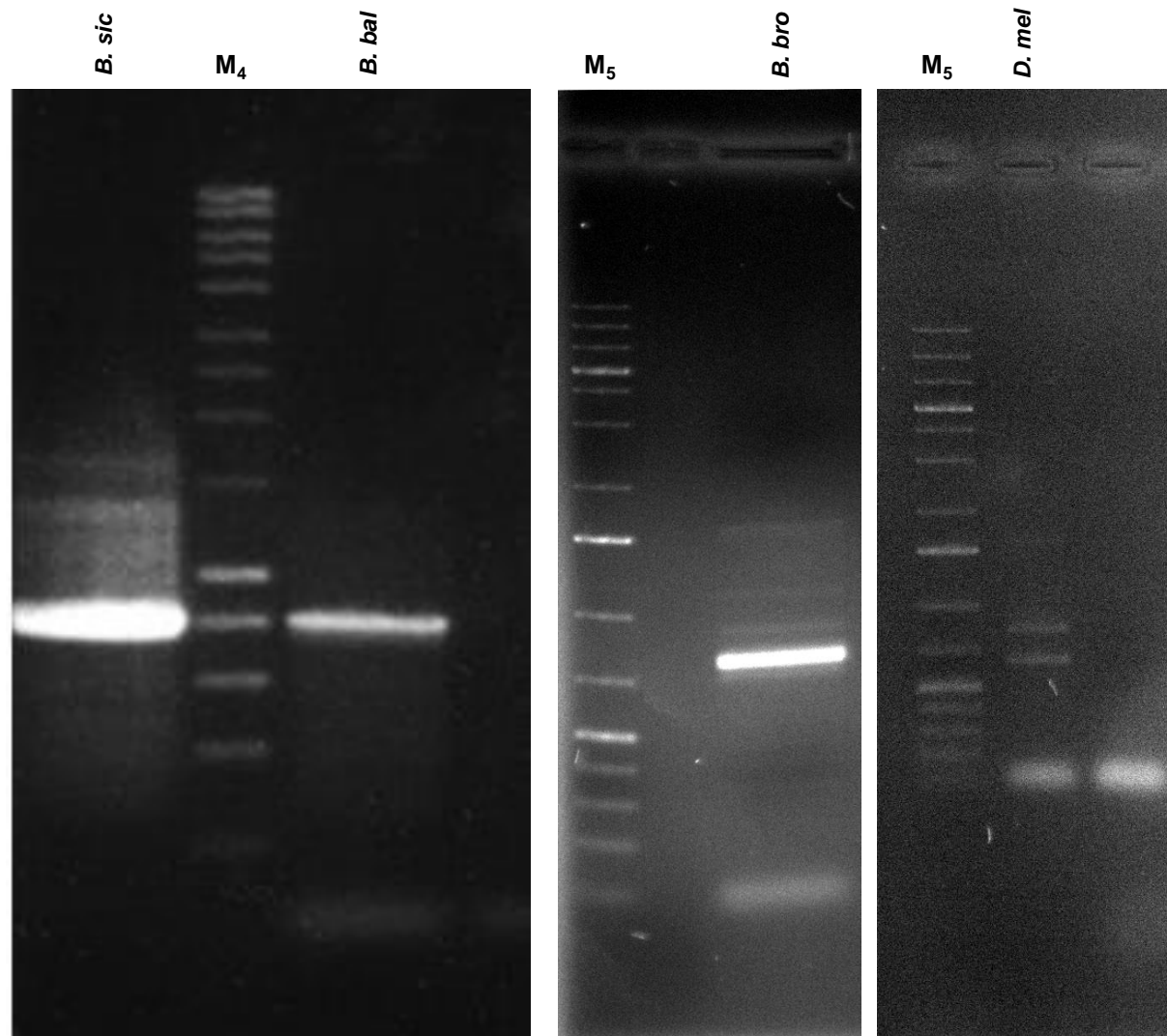

Fig. 1f (continuation)

Supplement: Supplementary file 7 — Supplementary Figure S7. [file 41598_2022_18051_MOESM7_ESM.pdf]
