# Supplementary material for: Identification and characterization of a new family of long satellite DNA, specific of true toads (Anura, Amphibia, Bufonidae)
Source: Sci Rep. 2022 Aug 17;12:13960. doi: 10.1038/s41598-022-18051-9 (PMC9385698; doi:10.1038/s41598-022-18051-9)
Supplement: Supplementary file 9 — Supplementary Table S2. [file 41598_2022_18051_MOESM9_ESM.pdf]

Groups		Range of distance variation within Groups	Average distance within Groups		Range of distance variation between Groups	Average distance between Groups	Net between Group mean distance
**Species**	*Bufo bufo*	0.0704-0.0176	0.0532±0.0051	Bbuf-Bspi	0.08214-0.01374	0.0497±0.0039	-0.0019±0.0004
	Bufo spinosus	0.0869-0.0000	0.0499±0.0038				
**Sex**	Male (M)	0.0711-0.0163	0.0481±0.0039	M-F	0.07326-0.00000	0.0460±0.0037	-0.0014±0.0002
	Female (F)	0.0718-0.0062	0.0468±0.0039				
**Population**	Italy (I)	0.0704-0.0176	0.0532±0.0051	I-S	0.08214-0.01374	0.0495±0.0040	-0.0020±0.0004
	Spain (S)	0.0869-0.0000	0.0498±0.0038	S-M	0.08145-0.01888	0.0500±0.0043	-0.0008±0.0005
	Morocco (M)	0.0716-0.0240	0.0517±0.0057	M-I	0.07347-0.02404	0.0508±0.0046	-0.0062±0.0007
**Experimental procedure**	Band (B)	0.0704-0.0176	0.0532±0.0051	B-PCR1	0.08214-0.02012	0.0518±0.0041	-0.0017±0.0004
	PCR1	0.0869-0.0189	0.0537±0.0042	PCR1-PCR2	0.07828-0.01754	0.0504±0.0040	0.0007±0.0004
	PCR2	0.0711-0.0000	0.0457±0.0039	PCR2-B	0.07326-0.01374	0.0477±0.0039	-0.0017±0.0005

Supplementary Table S2: Comparison of distances between *Bufo bufo* species group BamHI-800 sequences from different sex, population or experimental procedures. Standard error estimates were obtained by a bootstrap procedure (1000 replicates). Analyses were conducted using the Tamura-3 model of sequence evolution [73]. The rate of variation among sites was modelled with a gamma distribution (shape parameter = 2.8). All ambiguous positions were removed for each sequence pair (pairwise deletion option). There were a total of 819 positions in the final dataset. This analysis involved 54 (species: *Bufo bufo* (6), *Bufo spinosus* (48)), 32 (sex: male (13), female (19)), 54 (population: Italy (6), Morocco (5), Spain (43)) and 54 (method: band (6), PCR1 (23), PCR2 (25)) nucleotide sequences. Evolutionary analyses were conducted in MEGA X [68].
