# Supplementary material for: Identification and characterization of a new family of long satellite DNA, specific of true toads (Anura, Amphibia, Bufonidae)
Source: Sci Rep. 2022 Aug 17;12:13960. doi: 10.1038/s41598-022-18051-9 (PMC9385698; doi:10.1038/s41598-022-18051-9)
Supplement: Supplementary file 11 — Supplementary Table S4. [file 41598_2022_18051_MOESM11_ESM.pdf]

	Bufo bufo			*Bufo gargarizans*		
Chr.	RefSeq	N	n	RefSeq	N	n
1	NC_053389.1	74	305	NC_058080.1	40	98
2	NC_053390.1	73	151	NC_058081.1	19	95
3	NC_053391.1	37	210	NC_058082.1	78	264
4	NC_053392.1	2	84	NC_058083.1	2	127
5	NC_053393.1	17	1527	NC_058084.1	27	201
6	NC_053394.1	22	33	NC_058085.1	752	1215
7	NC_053395.1	17	80	NC_058086.1	4	13
8	NC_053396.1	1	5	NC_058087.1	0	0
9	NC_053397.1	0	3	NC_058088.1	0	1
10	NC_053398.1	207	328	NC_058089.1	0	6
11	NC_053399.1	79	283	NC_058090.1	14	20
-	unplaced	1004	2759	unplaced	40	65

Supplementary Table S4. Distribution of BamHI-800 hits in *Bufo bufo* and *Bufo gargarizans* genome assemblies. Total number of hits (n) and number of selected complete sequences (N) found after BLASTN searches on *Bufo bufo* (GCA_900303285.1) and *Bufo gargarizans* (GCA_014858855.1) genome assemblies.
