# Supplementary material for: Identification and characterization of a new family of long satellite DNA, specific of true toads (Anura, Amphibia, Bufonidae)
Source: Sci Rep. 2022 Aug 17;12:13960. doi: 10.1038/s41598-022-18051-9 (PMC9385698; doi:10.1038/s41598-022-18051-9)
Supplement: Supplementary file 16 — Supplementary Table S9. [file 41598_2022_18051_MOESM16_ESM.pdf]

MWM	Bands Size (in bp)
**M1**	1000, 900, 800, 700, 600, **500**, 450, 400, 350, 300, **250**, 200, 150, 100, 50
**M2**	10000, 8000, 6000, 5000, 4000, 3000, 2000, 1500, 1250, **1000**, 500
**M3**	1000, 900, 800, 700, 600, 500, 400, 300, 200, 150, 100
**M4**	**10000**, 8000, 6000, 5000, 4000, 3000, 2500, 2000, 1500, **1000**, 800, 600, 400, 200
**M5**	20000, 10000, 7000, **5000**, 4000, 3000, 2000, **1500**, 1000, 700, **500**, 400, 300, 200, 75

Supplementary Table S9: Molecular weight markers used in this work. M1: Ladder 50-1000 pb (dominion, mbl); M2: Ladder 500 bp-10 kb (dominion, mbl); M3: HyperLadder IV (Bioline, GmbH, Luckenwalde, Germany); M4: HyperLadder I (Bioline, GmbH, Luckenwalde, Germany); M5: GeneRuler 1kb Plus DNA Ladder (Fermentas (Vilnius, Lithuania)).
