# Supplementary material for: Identification and characterization of a new family of long satellite DNA, specific of true toads (Anura, Amphibia, Bufonidae)
Source: Sci Rep. 2022 Aug 17;12:13960. doi: 10.1038/s41598-022-18051-9 (PMC9385698; doi:10.1038/s41598-022-18051-9)
Supplement: Supplementary file 17 — Supplementary Information. [file 41598_2022_18051_MOESM17_ESM.pdf]

Supplementary Bibliography

1. Schmid, M. Chromosome Banding in Amphibia I. Constitutive Heterochromatin and Nucleolus Organizer Regions in *Bufo* and *Hyla*. *Chromosoma* **66**, 361–388 (1978).
2. Baldari, C. T. & Amaldi, F. DNA reassociation kinetics in relation to genome size in four amphibian species. *Chromosoma* **59**, 13–22 (1976).
3. Birstein, V. J. Structural Characteristics of Genome Organization in Amphibians: Differential Staining of Chromosomes and DNA Structure. *J. Mol. Evol.* **18**, 73–91 (1982).
4. Bozzoni, I. & Beccari, E. Clustered and interspersed repetitive DNA sequences in four amphibian species with different genome size. *Biochim. Biophys. Acta* **520**, 245–252 (1978).
5. Baldari, C. T. & Amaldi, F. Length and interspersion of repetitive and non repetitive DNA sequences in four Amphibian species with different genome sizes. *Chromosoma* **61**, 359–368 (1977).
6. Straus, N. A. Comparative DNA Renaturation Kinetics in Amphibians. *Proc. Natl. Acad. Sci. U. S. A.* **68**, 799–802 (1971).
7. Baldwin, L. & Macgregor, H. C. Centromeric satellite DNA in the newt *Triturus cristatus karelinii* and related species: Its distribution and transcription on lampbrush chromosomes. *Chromosoma* **92**, 100–107 (1985).
8. Cremisi, F., Vignali, R., Batistoni, R. & Barsacchi, G. Heterochromatic DNA in *Triturus* (Amphibia , Urodela). II. A centromeric satellite DNA. *Chromosom* **97**, 204–211 (1988).
9. Vignali, R. *et al.* Two dispersed highly repeated DNA families of *Triturus vulgaris meridionalis* (Amphibia, Urodela) are widely conserved among Salamandridae. *Chromosoma* **100**, 87–96 (1991).
10. Varley, J. M., Macgregor, H. C. & Barnett, L. Characterisation of a short, highly repeated and centromerically localised DNA sequence in crested and marbled newts of the genus *Triturus*. *Chromosoma* **100**, 15–31 (1990).
11. Batistoni, R., Nardi, I., Rebecchi, L., Nardone, M. & Demartis, A. A centromeric satellite DNA in the European plethodontid salamanders (Amphibia, Urodela). *Genome* **34**, 1007–1012 (1991).
12. Mizuno, S. *et al.* Variation of repetitive DNA and its phylogenetic relation in Hynobiidae (Caudata). *J. Hered.* **86**, 114–120 (1995).
13. Lam, B. S. & Carroll, D. Tandemly repeated DNA sequences from *Xenopus laevis*. I. Studies on sequence organization and variation in Satellite 1 DNA (741 base-pair repeat). *J. Mol. Biol.* **165**, 587–597 (1983).
14. Meyerhof, W., Tappeser, B., Korge, E. & Knöchel, W. Satellite DNA from *Xenopus laevis*: comparative analysis of 745 and 1037 base pair *Hin*d III tandem repeats. *Nucleic Acids Res*. **16**, 4465–4482 (1988).
15. Pasero, P., Sjakste, N., Blettry, C., Got, C. & Marilley, M. Long-range organization and sequence-directed curvature of *Xenopus laevis* satellite 1 DNA. *Nucleic Acids Res.* **21**, 4703–4710 (1993).
16. Picariello, O., Feliciello, I., Bellinello, R. & Chinali, G. S1 satellite DNA as a taxonomic marker in brown frogs: molecular evidence that *Rana graeca graeca* and *Rana graeca italica* are different species. *Genome* **45**, 63–70 (2002).
17. Feliciello, I., Picariello, O. & Chinali, G. The first characterisation of the overall variability of repetitive units in a species reveals unexpected features of satellite DNA. *Gene* 349, 153–**164** (2005).

18. Feliciello, I., Picariello, O. & Chinali, G. Intra-specific variability and unusual organization of the repetitive units in a satellite DNA from *Rana dalmatina*: Molecular evidence of a new mechanism of DNA repair acting on satellite DNA. *Gene* **383**, 81–92 (2006).
19. Cardone, D. E., Feliciello, I., Marotta, M., Rosati, C. & Chinali, G. A family of centromeric satellite DNAs from the European brown frog *Rana graeca italica*. *Genome* **40**, 774–781 (1997).
20. Bucci, S. et al. Rana/Pol III: a family of SINE-like sequences in the genomes of western Palearctic water frogs. *Genome* **42**, 504–11 (1999).
21. Marracci, S. et al. RrS1-like sequences of water frogs from Central Europe and around the Aegean Sea: chromosomal organization, evolution, possible function. *J. Mol. Evol.* **72**, 368–82 (2011).
22. Ragghianti, M., Bucci, S., Guerrini, F. & Mancino, G. Characterization of two repetitive DNA families (RrS1 and Rana/Pol III) in the genomes of Palaearctic green water frogs. *Ital. J. Zool.* **66**, 255–263 (1999).
23. Casola, C. et al. A hAT-related family of interspersed repetitive elements in genomes of western Palaearctic water frogs. *J. Zool. Syst. Evol. Res.* **42**, 234–244 (2004).
24. Ragghianti, M. et al. Molecular characterization of a centromeric satellite DNA in the hemiclonal hybrid frog *Rana esculenta* and its parental species. *Chromosom. Res*. **3**, 497–506 (1995).
25. Odierna, G. et al. Cytological and molecular analysis in the rare discoglossid species, *Alytes muletensis* (Sanchiz and Adrover 1977) and its bearing on archaeobatrachian phylogeny. *Chromosom. Res.* **8**, 435–442 (2000).
26. Odierna, G. et al. Chromosomal and molecular analysis of some repeated families in *Discoglossus* Otth, 1837 (Anura, Discoglossidae): taxonomic and phylogenetic implications. *Ital. J. Zool*. **66**, 273–283 (1999).
27. Picariello, O. et al. Characterization of two major satellite DNAs specific to the genus *Discoglossus* (Amphibia, Anura). *Ital. J. Zool*. **79**, 385–394 (2012).
28. Amor, N., Odierna, G., Chinali, G., Said, K. & Picariello, O. Unusual chromosomal distribution of a major satellite DNA from *Discoglossus pictus* (Amphibia, Anura). Cytogenet. *Genome Res*. **127**, 33–42 (2009).
